# Supplementary material for: Log BB Prediction Models Using TLC and HPLC Retention Values as Protein Affinity Data
Source: Pharmaceutics. 2024 Nov 30;16(12):1534. doi: 10.3390/pharmaceutics16121534 (PMC11678311; doi:10.3390/pharmaceutics16121534)

Table S1. Biological properties of 37 APIs

API	B1	LOGBB	B3	BB$_{vivo}$	CNS+/-	LactMed	LLL H	log U/D	M/P	PB	PhCharge
**acetazolamid**	-1,60	-1,29	0,52		1	1	2	-0,24	0,28	0,98	-1
**amitriptyline**	0,76	0,50	-0,45	0,89	1	1	2	-1,98	0,92	0,95	1
**bupivacaine**	0,16	0,03	1,25		1	1	2	-0,93	0,37	0,95	1
**chloroquine**	0,39	0,10	0,89		1	1	2	-3,27	2,65	0,55	2
**citalopram**	0,13	-0,03	-0,23		1	2	2	-2,37	1,03	0,80	1
**clomipramine**	0,79	0,44	-0,28		1	1	2	-2,26	1,13	0,98	1
**colchicine**	-0,93	-0,78	-1,99		1	2	4		0,95	0,39	0
**diltiazem**	-0,02	-0,40	-1,22		1	1	3	-1,74	0,99	0,75	1
**doxepin**	0,54	0,35	-0,18		1	1	5	-2,20	1,37	0,76	1
**duloxetine**	0,56	0,21	-0,65		1	1	3	-2,82	0,78	0,90	1
**eletriptan**	-0,19	-0,30	-0,84		1	1	3	-3,15	0,25	0,85	1
**ethambutol**	-0,86	-0,49	-0,52		0	1	2	-2,39	1,00	0,25	1
**fluvoxamine**	-0,14	-0,36	0,68		1	1	2	-2,19	1,33	0,79	1
**gabapentin**	-0,63	-0,47	-0,19		1	1	2	-0,29	1,00	0,03	0
**gentamycin**	-3,13	-2,65	-2,40		0	1	2	-2,57	0,36	0,15	5
**hydrochlorotiazide**	-1,62	-1,35	-2,29		0	1	2	-1,75	0,38	0,68	0
**itraconazole**	-0,66	-1,07	3,68		0	1	2	-0,73	1,14	1,00	0
**ketorolac**	-0,33	-0,40	0,23		1	1	2	-2,91	0,03	0,99	-1
**levetiracetam**	-0,93	-0,47	-1,02		1	1	3		1,00	0,10	0
**lincomycin**	-1,62	-1,47	-0,43		0		2	-1,58	0,15	0,70	1
**mesalazine**	-0,99	-0,79	-1,55		0	1	3	-5,30	2,69	0,43	-1
**minoxidil**	-1,00	-0,95	-0,18		1		3	-1,66	0,82	0,00	0
**nitrendipine**	-0,92	-1,22	-1,09		0	1	2	-4,41	0,30	0,99	0
**ofloxacin**	-0,66	-0,63	-1,97		0	1	2	-2,01	1,26	0,32	-1
**oxcarbamazepine**	-0,55	-0,47	-0,87		1		3	-6,53	0,50	0,40	0
**pefloxacin**	-0,52	-0,48	-1,44		0			-7,04	0,96	0,25	-1

**pregabalin**	-0,63	-0,47	-0,22		1	1	3	-0,57	0,55	0,00	0
**primidone**	-0,60	-0,38	-0,31		1	2	3	-5,06	0,72	0,70	0
**propylthiouracil**	-0,29	-0,11	-0,31		1	1	2	-0,43	0,17	0,82	0
**quinine**	-0,11	-0,18	-0,07		1	1	2	-2,08	0,23	0,70	1
**rifampicin**	-2,81	-2,98	-0,67		0	1	2	-2,24	0,40	0,89	1
**rimantadine**	0,26	0,13	0,43		1		3	-3,56	2,00	0,40	1
**timolol**	-0,85	-0,73	-0,06		0	1	2	-2,15	0,80	0,10	1
**tinidazole**	-1,35	-1,02	-0,65		1	1	3	-4,90	1,14	0,12	0
**tramadol**	0,01	0,02	-0,67		1	1	2	-2,41	2,30	0,20	1
**trimethoprim**	-1,33	-1,14	-1,90		1	1	2	-0,30	1,25	0,44	1
**warfarin**	-0,33	-0,47	-0,44		1	0	2	-2,70	0,00	0,99	-1

Table S2. Physicochemical properties of 37 APIs

API	a/b/n code[1]	DM	eH	eH-eL	eL[2]	HA	HD	log D	log P	MW[3]	pKa	PSA[4]	Sa[3]	V[3]
**acetazolamid**	0	0,89	-9,06	-9,13	0,69	5	2	-0,55	-0,26	2,22	7,44	11,50	4,69	2,9
**amitriptyline**	1	6,42	-9,9	-8,20	-17,01	1	0	3,15	4,41	2,77	9,18	0,32	3,61	1,56
**bupivacaine**	0	3,02	-9,03	-9,37	3,37	2	1	2,80	3,31	2,88	8,13	3,23	4,78	3,02
**chloroquine**	1	4,4	-8,6	-8,03	-5,71	3	1	1,87	4,41	3,20	10,47	2,82	5,94	3,11
**citalopram**	1	2,83	-7,98	-8,86	8,83	3	0	0,39	3,48	3,24	9,57	3,63	5,88	3,14
**clomipramine**	1	1,53	-8,49	-8,65	1,56	2	0	3,50	4,94	3,15	9,46	0,65	5,11	3,05
**colchicine**	0	5,95	-9,11	-8,52	-5,91	6	1	0,92	1,07	3,99		8,31	5,47	3,61
**diltiazem**	1	2,43	-8,71	-8,52	-1,86	4	0	2,10	4,73	4,15	8,94	5,91	6,17	3,96
**doxepin**	1	0,31	-9,11	-9,03	-0,81	2	0	2,08	3,84	2,79	9,40	1,25	4,69	2,81
**duloxetine**	1	0,96	-9,01	-8,63	-3,82	2	1	1,24	4,81	2,97	10,02	2,13	4,56	2,84
**eletriptan**	1	4,88	-8,5	-7,90	-5,96	3	1	0,36	2,98	3,83	10,35	5,32	4,69	3,57
**ethambutol**	1	1,11	-9,11	-11,45	23,39	4	4	-2,21	-0,29	2,04	9,59	6,45	4,95	2,18
**fluvoxamine**	1	3,9	-9,69	-8,87	-8,25	4	1	1,15	3,71	3,18	9,39	5,68	5,89	2,79
**gabapentin**	0	1,56	-9,35	-10,19	8,36	3	2	-1,31	1,08	1,71	7,50	6,33	2,38	1,7
**gentamycin**	1	4,31	-9,34	-11,20	18,62	12	8	-7,81	-2,04	4,78	9,77	19,97	4,71	4,25
**hydrochlorotiazide**	0	7,27	-9,64	-8,46	-11,83	5	3	-0,09	-0,02	2,98	8,95	11,84	3,85	2,01
**itraconazole**	0	6,11	-8,42	-7,65	-7,7	9	0	4,26	4,53	6,73	6,47	10,08	8,43	6,15
**ketorolac**	-1	2,14	-9,19	-8,83	-3,63	3	1	-0,95	2,68	2,55	4,29	5,93	3,87	2,39
**levetiracetam**	0	5,71	-9,8	-10,45	6,49	2	1	-0,67	-0,88	1,70		6,34	3,09	1,62
**lincomycin**	1	3,92	-8,5	-8,50	0,04	8	5	-0,48	0,72	4,07	8,78	12,60	6,35	3,89
**mesalazine**	-1	3,8	-8,18	-8,06	-1,23	4	3	-2,61	0,74	1,53	1,90	8,36	2,49	1,28
**minoxidil**	0	4,2	-8,07	-8,10	0,29	5	2	0,65	1,62	2,09	5,54	9,36	3,16	1,96
**nitrendipine**	0	6,92	-9,09	-8,00	-10,94	5	1	3,50	3,81	3,60	2,79	11,05	5,3	2,98
**ofloxacin**	-1	4,68	-8,72	-7,82	-8,97	7	1	-0,65	1,85	3,61	5,19	7,33	4,74	3,07
**oxcarbamazepine**	0	3,46	-9,31	-8,84	-4,73	2	1	1,25	1,66	2,52	13,73	6,34	3,02	2,26
**pefloxacin**	-1	9,41	-9,01	-7,97	-10,45	6	1	0,52	1,92	3,33	0,16	6,41	4,66	2,91
**pregabalin**	0	1,67	-9,53	-10,46	9,29	3	2	-1,38	1,09	1,59	7,77	6,33	3,54	1,65
**primidone**	0	2,84	-9,78	-9,77	-0,15	2	2	0,40	0,83	2,18	12,26	5,82	3,19	2,02

**propylthiouracil**	0	5,57	-9,2	-7,93	-12,71	1	2	1,18	1,15	1,70	7,63	4,11	3,32	1,51
**quinine**	1	1,73	-9,55	-9,54	-0,14	4	1	1,58	2,82	3,24	9,28	4,56	4,22	3,06
**rifampicin**	-1					14	6	-0,46	2,05	8,23	4,96	22,02		
**rimantadine**	1	1,22	-9,33	-12,28	29,54	1	1	0,08	3,31	1,79	10,76	2,60	2,43	1,91
**timolol**	1	3,06	-9,17	-8,05	-11,24	7	2	-1,39	1,28	3,16	9,35	7,97	4,95	2,88
**tinidazole**	0	2,34	-10,52	-9,24	-12,82	5	0	-0,27	-0,29	2,47	2,30	9,78	4,41	2
**tramadol**	1					3	1	0,36	2,32	2,63	9,61	3,27		
**trimethoprim**	0	2,09	-8,8	-8,68	-1,22	7	2	0,58	0,59	2,90	6,90	10,55	4,14	2,63
**warfarin**	-1	4,12	-7,38	-6,05	-13,35	3	1	-0,90	3,13	3,08	4,50	6,36	4,01	2,76

1 – code describing the acid-base properties: (-1) - acids; (0) - neutrals; (1) - bases

2 – multiplied by 10

3 – divided by 100

4 – divided by 10

Table S3. Chromatographic data and their derivatives obtained from NP TLC and RP TLC experiments

API	C_{NP}*	NP**	NP/C	NP/PSA	C_{RP}*	RP**	RP/C	RP/PSA	NP/B2	RP/B2	NP/PB	RP/PB	NP/logP	RP/logP
**acetazolamid**	0,94	0,94	1,00	0,817	0,97	0,96	0,99	0,834	-0,727	-0,742	0,959	0,980	-3,615	-3,692
**amitriptyline**	0,32	0,31	0,97	9,568	0,67	0,63	0,94	19,444	0,626	1,272	0,326	0,663	0,070	0,143
**bupivacaine**	0,67	0,67	1,00	2,072	0,74	0,75	1,01	2,319	22,666	25,372	0,705	0,789	0,202	0,227
**chloroquine**	0,17	0,17	1,00	0,604	0,40	0,30	0,75	1,065	1,763	3,111	0,309	0,545	0,039	0,068
**citalopram**	0,30	0,29	0,97	0,800	0,62	0,60	0,97	1,655	-8,745	-18,094	0,363	0,750	0,083	0,172
**clomipramine**	0,33	0,32	0,97	4,938	0,59	0,59	1,00	9,105	0,722	1,331	0,327	0,602	0,065	0,119
**colchicine**	0,79	0,77	0,97	0,927	0,91	0,91	1,00	1,095	-0,984	-1,163	1,974	2,333	0,720	0,850
**diltiazem**	0,49	0,49	1,00	0,829	0,76	0,72	0,95	1,219	-1,230	-1,808	0,653	0,960	0,104	0,152
**doxepin**	0,30	0,31	1,03	2,486	0,58	0,54	0,93	4,330	0,892	1,554	0,411	0,715	0,081	0,141
**duloxetine**	0,32	0,33	1,03	1,552	0,57	0,53	0,93	2,493	1,595	2,562	0,367	0,589	0,069	0,110
**eletriptan**	0,29	0,28	0,97	0,527	0,62	0,62	1,00	1,166	-0,922	-2,041	0,329	0,729	0,094	0,208
**ethambutol**	0,19	0,21	1,11	0,325	0,97	0,95	0,98	1,472	-0,433	-1,957	0,840	3,800	-0,724	-3,276
**fluvoxamine**	0,34	0,32	0,94	0,563	0,62	0,62	1,00	1,091	-0,883	-1,711	0,408	0,790	0,086	0,167
**gabapentin**	0,91	0,91	1,00	1,437	0,66	0,66	1,00	1,042	-1,952	-1,416	30,333	22,000	0,843	0,611
**gentamycin**	0,96	0,94	0,98	0,471	0,91	0,90	0,99	0,451	-0,355	-0,340	6,267	6,000	-0,461	-0,441
**hydrochlorotiazide**	0,96	0,96	1,00	0,811	0,97	0,96	0,99	0,811	-0,713	-0,713	1,414	1,414	-48,000	-48,000
**itraconazole**	0,96	0,97	1,01	0,962	0,86	0,84	0,98	0,833	-0,910	-0,788	0,972	0,842	0,214	0,185
**ketorolac**	0,92	0,91	0,99	1,535	0,96	0,96	1,00	1,619	-2,265	-2,389	0,919	0,970	0,340	0,358
**levetiracetam**	0,92	0,92	1,00	1,451	0,92	0,92	1,00	1,451	-1,968	-1,968	9,200	9,200	-1,045	-1,045
**lincomycin**	0,91	0,91	1,00	0,722	0,94	0,94	1,00	0,746	-0,620	-0,640	1,300	1,343	1,264	1,306
**mesalazine**	0,94	0,93	0,99	1,113	0,99	0,97	0,98	1,161	-1,178	-1,228	2,163	2,256	1,257	1,311
**minoxidil**	0,43	0,42	0,98	0,449	0,80	0,82	1,03	0,876	-0,442	-0,862			0,259	0,506
**nitrendipine**	0,97	0,97	1,00	0,878	0,86	0,86	1,00	0,779	-0,795	-0,705	0,980	0,869	0,255	0,226
**ofloxacin**	0,24	0,23	0,96	0,314	0,54	0,50	0,93	0,682	-0,367	-0,799	0,719	1,563	0,124	0,270
**oxcarbamazepine**	0,91	0,90	0,99	1,420	0,91	0,91	1,00	1,435	-1,926	-1,947	2,250	2,275	0,542	0,548
**pefloxacin**	0,30	0,29	0,97	0,452	0,76	0,70	0,92	1,092	-0,606	-1,463	1,160	2,800	0,151	0,365
**pregabalin**	0,76	0,76	1,00	1,200	0,41	0,41	1,00	0,648	-1,630	-0,880			0,697	0,376
**primidone**	0,90	0,93	1,03	1,598	0,93	0,97	1,04	1,667	-2,421	-2,525	1,329	1,386	1,120	1,169

**propylthiouracil**	0,94	0,94	1,00	2,285	0,93	0,93	1,00	2,261	-8,462	-8,372	1,146	1,134	0,817	0,809
**quinine**	0,30	0,29	0,97	0,636	0,66	0,64	0,97	1,404	-1,590	-3,508	0,414	0,914	0,103	0,227
**rifampicin**	0,73	0,77	1,05	0,350	0,95	0,94	0,99	0,427	-0,259	-0,316	0,865	1,056	0,376	0,459
**rimantadine**	0,92	0,92	1,00	3,536	0,95	0,95	1,00	3,651	7,040	7,270	2,300	2,375	0,278	0,287
**timolol**	0,34	0,32	0,94	0,401	0,97	0,97	1,00	1,216	-0,439	-1,331	3,200	9,700	0,250	0,758
**tinidazole**	0,89	0,88	0,99	0,900	0,94	0,93	0,99	0,951	-0,865	-0,914	7,333	7,750	-3,034	-3,207
**tramadol**	0,31	0,31	1,00	0,948	0,63	0,64	1,02	1,957	13,025	26,891	1,550	3,200	0,134	0,276
**trimethoprim**	0,57	0,59	1,04	0,559	0,84	0,83	0,99	0,787	-0,517	-0,727	1,341	1,886	1,000	1,407
**warfarin**	0,99	0,99	1,00	1,557	0,87	0,83	0,95	1,305	-2,104	-1,764	1,000	0,838	0,316	0,265

* Rf value from unimpregnated NP or RP plate (control)

** Rf value from NP or RP plate impregnated with 2 mg/mL BSA solution

Table S4. Chromatographic data and their derivatives obtained from the HPLC experiments

API	k_{HSA}	log k_{HSA}	log k_{HSA}/B2	log k_{HSA}/PB	log k_{HSA}/log P	k_{IAM}	log k_{IAM}
**acetazolamid**	0,74	-0,13	-0,575	0,760	-2,863	2,90	0,46
**amitriptyline**	5,87	0,77	11,854	6,178	1,331	1,56	0,19
**bupivacaine**	1,18	0,07	40,084	1,247	0,358	3,02	0,48
**chloroquine**	18,05	1,26	187,127	32,812	4,092	3,11	0,49
**citalopram**	2,67	0,43	-80,418	3,333	0,766	3,14	0,50
**clomipramine**	9,09	0,96	20,510	9,278	1,841	3,05	0,48
**colchicine**	0,28	-0,55	-0,362	0,726	0,265	3,61	0,56
**diltiazem**	1,74	0,24	-4,362	2,316	0,367	3,96	0,60
**doxepin**	4,25	0,63	12,220	5,624	1,106	2,81	0,45
**duloxetine**	8,16	0,91	39,435	9,063	1,696	2,84	0,45
**eletriptan**	3,78	0,58	-12,429	4,441	1,267	3,57	0,55
**ethambutol**	1,73	0,24	-3,557	6,906	-5,953	2,18	0,34
**fluvoxamine**	0,25	-0,60	-0,696	0,321	0,068	2,79	0,45
**gabapentin**	0,15	-0,84	-0,312	4,843	0,135	1,70	0,23
**gentamycin**	1,50	0,18	-0,568	10,028	-0,737	4,25	0,63
**hydrochlorotiazide**	0,61	-0,22	-0,452	0,897	-30,451	2,01	0,30
**itraconazole**	9,65	0,98	-9,055	9,668	2,130	6,15	0,79
**ketorolac**	9,65	0,98	-24,017	9,747	3,601	2,39	0,38
**levetiracetam**	0,07	-1,16	-0,149	0,696	-0,079	1,62	0,21
**lincomycin**	0,82	-0,08	-0,560	1,175	1,142	3,89	0,59
**mesalazine**	0,18	-0,73	-0,234	0,430	0,250	1,28	0,11
**minoxidil**	0,40	-0,40	-0,422		0,248	1,96	0,29
**nitrendipine**	3,55	0,55	-2,906	3,581	0,931	2,98	0,47
**ofloxacin**	6,62	0,82	-10,566	20,673	3,576	3,07	0,49
**oxcarbamazepine**	0,40	-0,40	-0,846	0,988	0,238	2,26	0,35
**pefloxacin**	12,43	1,09	-25,980	49,720	6,474	2,91	0,46
**pregabalin**	0,14	-0,87	-0,291		0,124	1,65	0,22
**primidone**	0,22	-0,66	-0,569	0,312	0,263	2,02	0,31

**propylthiouracil**	0,33	-0,48	-2,950	0,400	0,285	1,51	0,18
**quinine**	2,59	0,41	-14,194	3,699	0,918	3,06	0,49
**rifampicin**	0,03	-1,53	-0,010	0,034	0,015	8,37	0,92
**rimantadine**	0,16	-0,80	1,222	0,399	0,048	1,91	0,28
**timolol**	0,13	-0,88	-0,179	1,304	0,102	2,88	0,46
**tinidazole**	0,12	-0,92	-0,118	0,997	-0,413	2,00	0,30
**tramadol**	1,08	0,03	45,221	5,381	0,464	3,90	0,59
**trimethoprim**	0,85	-0,07	-0,749	1,943	1,449	2,63	0,42
**warfarin**	13,86	1,14	-29,454	14,001	4,428	2,76	0,44

Table S5. Structures of 37 APIs

1. acetazolamid

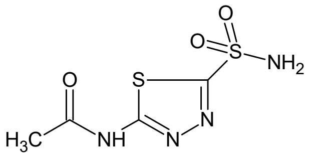

2. amitriptyline

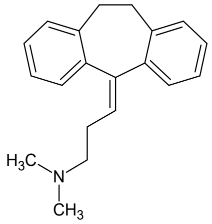

3. bupivacaine

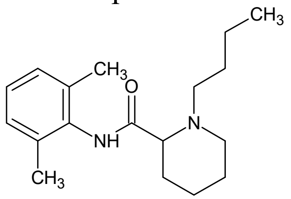

4. chloroquine

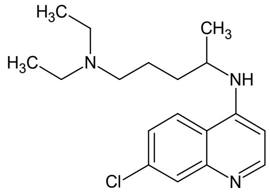

5. citalopram

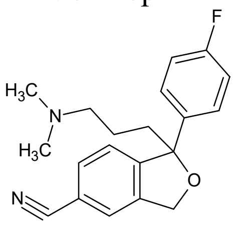

6. clomipramine

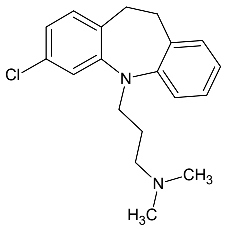

7. colchicine

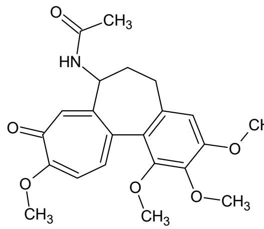

8. diltiazem

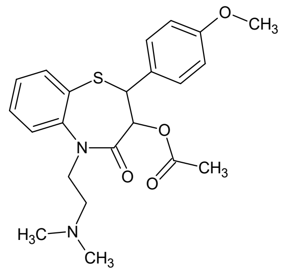

9. doxepin

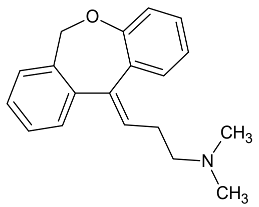

10. duloxetine

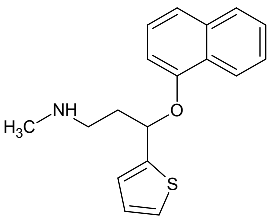

11. eletriptan

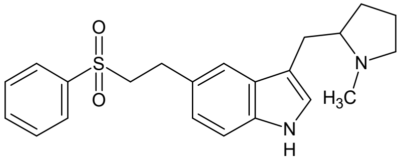

12. ethambutol

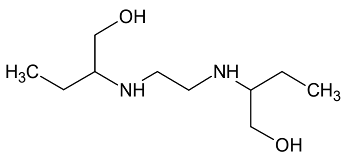

13. fluvoxamine

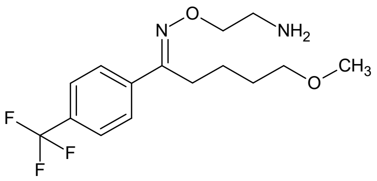

14. gabapentin

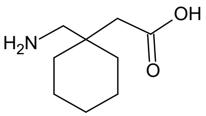

15. gentamicin

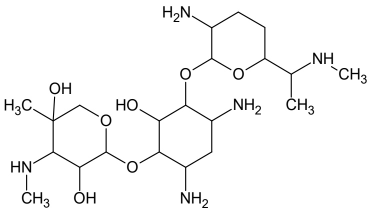

16. hydrochlorotiazide

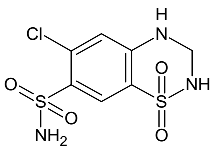

17. itraconazole

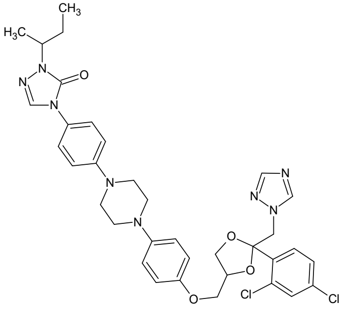

18. ketorolac

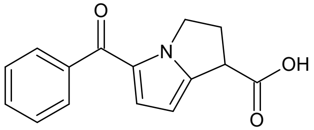

19. levetiracetam

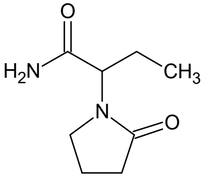

20. lincomycin

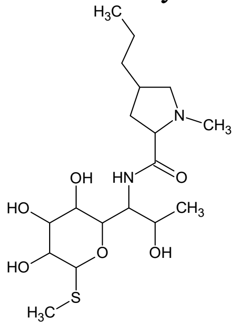

21. mesalazine

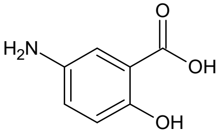

22. minoxidil

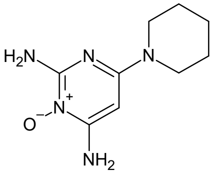

23. nitrendipine

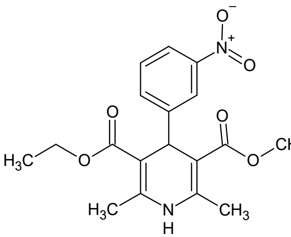

24. ofloxacin

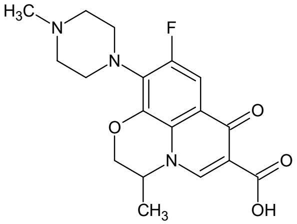

25. oxcarbamazepine

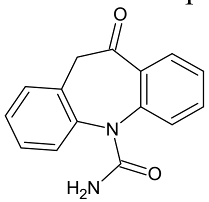

26. pefloxacin

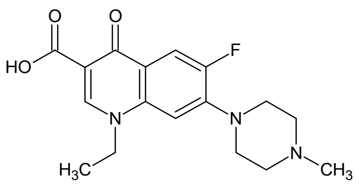

27. pregabalin

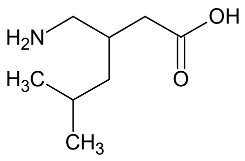

28. primidone

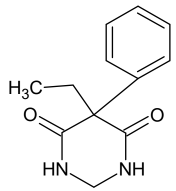

29. propylthiouracil

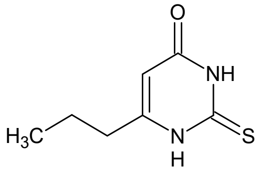

30. quinine

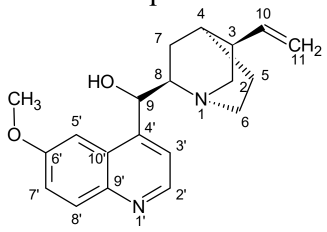

31. rifampicin

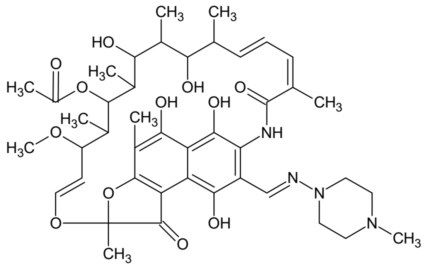

32. rimantadine

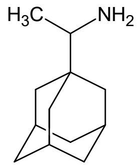

33. timolol

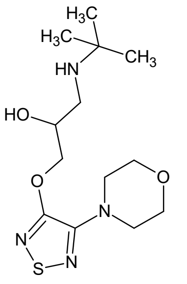

34. tinidazole

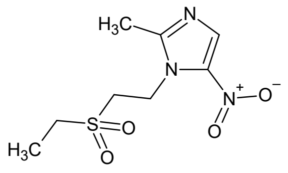

35. tramadol

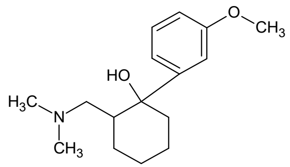

36. trimethoprim

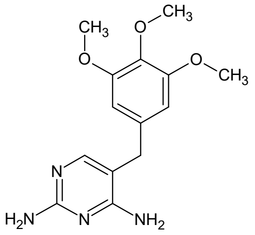

37. warfarin

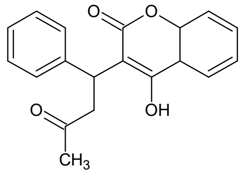

Table S6. Dataset of 181 APIs

	APIs	CNS+/−	LOGBB	LOGBB > −0.9	LOGBB > −0.52	log U/D	PB	PB_{code}	NP	NP/C	RP	RP/C	logk(HSA)	MW*	HD	HA	HA+HD	DM	eH	eL**	eH−eL	logD	Sa*	V*	logP	logk(IAM)
**1**	**acebutolol**	0	−0.86	1	0	−2.2	0.26	4	0.46	1.02	0.82	1.05	0.05	3.36	3	5	8	2.65	−9.19	−5.11	−8.68	0.26	6.33	3.29	−0.33	0.70
**2**	**aceclofenac**		−0.66	1	0	−4.6	0.99	1	0.90	1.01	0.93	1.00	1.75	3.54	2	5	7	1.93	−8.85	−2.68	−8.58	0.44	4.44	2.84	3.54	0.42
**3**	**acenocumarol**	1	−1.20	0	0	−2.7	0.987	1	0.98	0.99	0.92	0.99	1.27	3.53	1	7	8	8.94	−9.74	−14.71	−8.27	0.34	4.66	2.96	2.68	−0.27
**4**	**acetazolamid**	1	−1.29	0	0	−0.24	0.98	1	0.94	1.00	0.96	0.99	−0.13	2.77	2	5	7	0.89	−9.06	0.69	−9.13	−0.55	4.69	2.9	2.36	
**5**	**acetylsalicylic acid**	1	−0.47	1	1	−3.72	0.995	1	0.90	1.01	0.99	1.01	−0.51	1.80	1	4	5	5.18	−9.78	−6.93	−9.08	−1.89	3.05	1.52	1.30	−1.52
**6**	**aciclovir**		−1.21	0	0	−2.09	0.21	4	0.73	0.99	0.98	0.99	−0.72	2.25	4	8	12	5.69	−8.76	−4.89	−8.27	−1.76	3.59	1.86	−1.45	−1.04
**7**	**allopurinnol**	1	−0.51	1	1	−2			0.88	0.99	0.93	0.96	−0.82	1.36	2	5	7	8.84	−8.77	−10.78	−7.69	−0.95	2.06	1.08	−0.93	
**8**	**alprazolam**	1	−0.06	1	1	−4.83	0.8	2	0.86	1.01	0.78	0.98	−0.05	3.09	0	4	4	6.16	−9.51	−1.42	−9.36	2.5	2.94	2.69	4.68	0.50
**9**	**amiodarone**	1	−0.14	1	1	−2.17	0.96	1	0.6	1.09	0.34	1.03	2.09	6.45	0	3	3	5.30	−7.46	−33.48	−4.11	6.94	5.21	4.46	3.01	2.00
**10**	**amitriptyline**	1	0.50	1	1	−1.98	0.95	1	0.31	0.97	0.63	0.94	0.77	2.22	0	1	1	6.42	−9.9	−17.01	−8.20	3.15	3.61	1.56	0.44	
**11**	**amlodipine**	0	−1.05	0	0	−1.77	0.975	1	0.56	1.00	0.68	1.11	0.74	4.09	2	5	7	2.88	−8.66	−3.35	−8.32	2.59	5.92	3.62	−1.51	2.00
**12**	**amoxilcillin**	0	−1.99	0	0	−4.76	0.2	4	0.68	1.00	0.98	0.99	−0.03	3.65	5	8	13	4.32	−9.20	−3.31	−8.87	−2.43	4.88	3.07	−0.15	−1.32
**13**	**astemizol**	1	−0.05	1	1	−1.48	0.967	1	0.61	0.98	0.62	0.95	1.12	4.59	1	5	6	1.71	−7.83	−3.07	−7.52	4.08	6.02	4.22	0.71	1.42
**14**	**atenolol**	0	−0.81	1	0	−2.23			0.33	0.97	0.56	0.97	−0.11	2.66	4	5	9	6.13	−9.33	2.61	−9.59	−1.74	2.95	2.34	0.83	−0.10
**15**	**atorvastatin**	0	−1.24	0	0	−2.91	0.98	1	0.9	1.00	0.89	1.11	0.54	5.63	4	5	9	2.22	−9.00	−1.40	−8.86	1.11	6.75	5.21	1.54	0.92
**16**	**atropine**	1	−0.25	1	1	−2.78	0.18	4	0.26	0.96	0.72	0.99	0.09	2.89	1	4	5	2.48	−9.42	0.41	−9.46	−0.94	4.24	2.79	1.71	0.59
**17**	**azithromycin**	0	−2.33	0	0	−1.39	0.29	4	0.63	1.00	0.24	1.00	−0.64		5	13	18				0.00	3.18			2.44	−1.20
**18**	**betahistine**	1	0.15	1	1	−2.55			0.16	0.94	0.33	1.06		1.36	1	2	3	2.91	−9.24	−0.60	−9.18	−2.18	3.27	1.44	1.18	−0.05
**19**	**betaxolol**	0	−0.26	1	1	−2.23	0.5	3	0.65	0.98	0.25	0.93	0.20	3.07	2	4	6	1.20	−8.97507	4.43	−9.42	0.93	5.89	3.15	1.00	1.19
**20**	**bilastine**		−0.71	1	0	−0.61	0.87	2	0.44	0.96	0.72	0.95	−0.17	4.64	2	6	8	2.69	−8.82	−1.22	−8.70	1.97	6.65	4.54	2.10	−0.26
**21**	**biperiden**	1	0.17	1	1	−2.06	0.6	3	0.65	0.96	0.85	0.98	−0.24	3.11	1	2	3	2.30	−9.16	3.22	−9.48	1.68	4.11	3.20	2.71	1.62
**22**	**bisoprolol**	0	−0.41	1	1	−2.22	0.3	4	0.66	0.98	0.56	0.98	0.03	3.25	2	5	7	1.93	−9.09	3.27	−9.42	0.4	7.05	3.31	2.13	0.83
**23**	**bromazepam**	1	−0.32	1	1	−4.19	0.7	2	0.90	1.03	0.81	0.98	−0.20	3.16	1	4	5	2.87	−9.13	−1.07	−9.02	1.65	2.67	2.36	2.59	0.34
**24**	**bromocriptine**	0	−1.34	0	0	−0.76	0.93	1	0.96	1.01	0.70	1.01	0.91	6.69	3	10	13	4.25	−8.92	−9.58	−7.96	5.07	6.06	5.64	4.48	1.10
**25**	**bupivacaine**	1	0.03	1	1	−0.93	0.95	1	0.67	1.00	0.75	1.01	0.07	2.88	1	2	3	3.02	−9.03	3.37	−9.37	2.80	4.78	3.02	3.82	
**26**	**buspirone**	1	−0.57	1	1	−0.52	0.95	1	0.58	1.01	0.69	0.99	−0.05	3.86	0	7	7	4.32	−8.74	−1.56	−8.58	3.35	5.19	3.71	1.18	0.84
**27**	**caffeine**	1	−0.31	1	1	−6.68	0.3	4	0.82	1.00	0.90	0.99	−0.64	1.94	0	6	6	3.92	−9.00	−5.36	−8.47	−0.13	3.41	1.68	−1.06	−0.75
**28**	**capecitabine**	1	−1.38	0	0	−1.79	0.6	3	0.92	1.00	0.89	0.99	−0.52	3.59	3	9	12	4.97	−9.72	−13.37	−8.38	−0.73	5.37	3.04	0.53	0.04
**29**	**captopril**	1	−0.37	1	1	−3.61	0.275	4	0.74	0.96	0.99	1.00	−0.82	2.17	2	3	5	1.87	−9.35	1.05	−9.45	−3.15	3.63	1.92	0.30	−2.19

**30**	**carbamazepine**	1	−0.19	1	1	−6.74	0.76	2	0.95	0.99	0.84	1.01	0.04	2.36	2	3	5	3.25	−9.05	−5.19	−8.53	2.67	2.85	2.19	2.10	0.18
**31**	**carbegoline**	1	−0.60	1	1	−2.21	0.41	3	0.68	1.00	0.68	1.00	0.51	4.24	2	7	9	3.58	−8.39	−2.38	−8.15	−0.19	6.41	4.10	0.29	1.23
**32**	**carvedilol**	0	−0.66	1	0	−1.04	0.98	1	0.9	1.23	0.61	1.03	1.16	4.20	3	5	8	3.15	−8.56	−2.09	−8.35	3.29	5.49	3.97	−2.04	
**33**	**cefuroxime**	0	−2.64	0	0	−4.61	0.5	3	0.98	1.01	0.87	0.99	−0.64	4.24	12	4	16	2.73	−8.76	−13.17	−7.44	2.91	4.26	3.75	−2.08	0.23
**34**	**celecoxib**	1	−0.70	1	0	−2.48	0.97	1	0.99	1.01	0.75	1.00	1.13	3.81	1	3	4	4.09	−9.93	−14.21	−8.51	4.21	5.10	2.97	1.02	1.70
**35**	**celiprolol**		−0.91	0	0	−2.3	0.275	4	0.46	1.02	0.79	1.05	0.05	3.65	3	5	8	2.01	−9.24	−4.56	−8.78	0.22	6.62	3.59	−0.45	0.77
**36**	**cephalexin**	0	0.03	1	1	−4.08	0.14	4	0.9	1.00	0.99	1.00	−0.80	3.47	3	5	8	5.35	−9.59	−6.25	−8.97	−2.44	4.24	2.95	−1.31	
**37**	**cetrizine**	1	−0.30	1	1	−3.74	0.93	1	0.65	1.01	0.82	0.99	0.19	3.75	1	5	6	3.56	−8.97	−2.20	−8.75	−1.13	5.01	3.37	2.11	0.02
**38**	**chloramphenikol**	0	−1.30	0	0	−3.83	0.55	3	0.97	0.98	0.89	1.00	−0.21	3.23	3	7	10	−9.08	−10.34	−13.37	−9.01	1.02	4.50	2.44	−0.25	0.03
**39**	**chloroquine**	1	0.10	1	1	−3.27	0.55	3	0.17	1.00	0.30	0.75	1.26	3.05	1	3	4	4.4	−8.6	−5.71	−8.03	1.87	5.94	3.11	3.11	
**40**	**chlorpromazine**	1	0.04	1	1	−2.21	0.9	2	0.43	0.99	0.50	1.02	1.12	3.19	0	2	2	7.13	−6.83	0.74	−6.90	3.26	3.04	2.62	3.77	1.77
**41**	**chlortalidone**	1	−1.34	0	0	−2.37	0.75	2	0.96	0.99	0.92	1.01	−0.07	3.19	4	6	10	4.04	−9.45	−7.73	−8.68	−0.74	3.79	2.51	−0.63	0.12
**42**	**cimetidine**	0	−1.28	0	0	−0.13	0.17	4	0.67	1.05	0.87	0.99	−0.17	2.52	3	6	9	11.01	−8.75	−2.76	−8.48	−0.25	2.94	2.29	−0.59	−0.58
**43**	**ciprofloxacin**		−0.62	1	0	−0.36	0.3	4	0.11	1.00	0.21	0.55	−0.72	331.35	2	6	8	6.93	−8.72	−7.85	−7.94	−0.85	4.25	2.84	−1.85	0.09
**44**	**cisapride**	1	−0.83	1	0	−0.27	0.975	1	0.63	0.98	0.62	0.95	0.69	4.66	3	7	10	1.68	−8.72	−1.86	−8.54	2.6	6.66	4.11	2.25	1.76
**45**	**citalopram**	1	−0.03	1	1	−2.37	0.80	2	0.29	0.97	0.60	0.97	0.43	3.24	0	3	3	2.83	−7.98	8.83	−8.86	0.39	5.88	3.14	0.87	
**46**	**clarithromycin**	0	−2.38	0	0	−0.96	0.7	2	0.99	0.99	0.78	0.94	−0.09		4	14	18				0.00	2.33			3.16	−1.02
**47**	**clindamycin**	0	−1.15	0	0	−1.53	0.93	1	0.66	0.96	0.25	1.00	0.13		4	7	11				0.00	0.48			1.59	1.05
**48**	**clobazam**	1	−0.10	1	1	−1.39	0.85	2	0.95	1.00	0.80	1.01	0.00	3.01	0	4	4	4.18	−9.21	−5.52	−8.66	1.59	4.02	2.58	−1.40	0.41
**49**	**clomipramine**	1	0.44	1	1	−2.26	0.98	1	0.32	0.97	0.59	1.00	0.96	3.15	0	2	2	1.53	−8.49	1.56	−8.65	3.50	5.11	3.05	0.78	
**50**	**clonidine**	1	−0.04	1	1	−0.9	0.3	3	0.50	1.02	0.68	0.93	0.13	2.30	2	3	5	3.51	−8.90	−4.25	−8.48	−0.68	3.10	1.86	0.28	0.45
**51**	**clorazepate**	1	−1.09	0	0	−1.9	0.975	1	0.94	1.00	0.81	1.00	0.60	3.15	4	6	10	3.51	−9.12	−1.13	−9.01	1.55	2.91	2.57	2.75	0.69
**52**	**clozapine**	1	0.50	1	1	−0.13	0.99	1	0.46	1.01	0.57	1.00	0.84	2.87	0	1	1	1.13	−8.78	−2.69	−8.51	4.86	3.81	2.90	1.77	1.71
**53**	**colchicine**	1	−0.78	1	0		0.39	3	0.77	0.97	0.91	1.00	−0.55	3.99	1	6	7	5.95	−9.11	−5.91	−8.52	0.92	5.47	3.61	−2.65	
**54**	**cyproheptadine**	1	0.10	1	1	−1.75	0.975	1	0.43	0.94	0.47	1.04	0.99	2.65	1	3	4	1.98	−8.63	2.03	−8.83	2.44	3.89	2.66	1.16	1.38
**55**	**desloratidine**	1	0.15	1	1	−3.07	0.845	2	0.35	0.97	0.58	1.00	0.78	3.11	1	2	3	3.04	−9.13	−2.94	−8.83	4.1	3.75	2.88	0.07	1.70
**56**	**diazepam**	1	0.02	1	1	−3.8	0.98	1	0.95	0.99	0.77	1.00	0.55	2.85	0	3	3	3.57	−9.06	−1.00	−8.96	2.91	2.80	2.50	3.01	0.71
**57**	**digoxin**	0	−2.70	0	0	−6.3	0.25	4	0.77	0.96	0.85	1.01	−0.68	7.81	6	14	20	5.58	−8.45	−11.68	−7.28	0.85	7.66	7.05	2.67	0.57
**58**	**dihydroergotamine**	0	−1.34	0	0	−0.02	0.93	2	0.75	1.04	0.78	1.00	−0.64	4.78	3	6	9	3.90	−8.60	−1.05	−8.50	2.05	5.13	4.33	−2.11	
**59**	**diltiazem**	1	−0.40	1	1	−1.74	0.75	2	0.49	1.00	0.72	0.95	0.24	4.29	0	4	4	2.43	−8.71	−1.86	−8.52	2.10	6.17	3.96	−1.11	
**60**	**diphenhydramin**	1	0.35	1	1	−1.56	0.985	1	0.33	0.77	0.54	0.93	0.27	2.55	0	2	2	0.52	−9.18	2.36	−9.41	2.29	4.92	2.65	2.09	0.56
**61**	**doxazosin**	1	−1.42	0	0	−0.68	0.98	1	0.81	1.01	0.73	0.97	0.74	4.09	1	9	10	4.02	−8.67	−11.34	−7.54	0.6	4.93	3.46	2.14	−0.66
**62**	**doxepin**	1	0.35	1	1	−2.20	0.76	2	0.31	1.03	0.54	0.93	0.63	2.79	0	2	2	0.31	−9.11	−0.81	−9.03	2.08	4.69	2.81	0.98	

**63**	**doxycycline**	0	−2.36	0	0	−0.47	0.9	2	0.05	0.75	0.09	1.13	0.23	4.44	7	10	17	11.15	−9.00	−11.51	−7.85	−3.32	4.43	3.68	−1.86	0.24
**64**	**drotaverine**	1	−0.24	1	1	−1.03	0.875	2	0.59	0.97	0.58	1.05	0.28	3.98	1	5	6	3.56	−8.36	−3.63	−7.99	6.12	6.39	3.89	−1.51	1.49
**65**	**duloxetine**	1	0.21	1	1	−2.82	0.90	1	0.33	1.03	0.53	0.93	0.91	2.97	1	2	3	0.96	−9.01	−3.82	−8.63	1.24	4.56	2.84	0.04	
**66**	**eletriptan**	1	−0.30	1	1	−3.15	0.85	2	0.28	0.97	0.62	1.00	0.58	3.82	1	3	4	4.88	−8.5	−5.96	−7.90	0.36	4.69	3.57	−0.35	
**67**	**enalapril**	0	−0.99	0	0	−4.05	0.55	3	0.33	0.98	0.58	0.97	−0.47	3.75	2	7	9	3.46	−9.37	0.20	−9.38	−2.35	4.19	3.48	2.30	−1.29
**68**	**eplerenone**	1	−0.72	1	0		0.5	3	0.94	1.00	0.85	1.01	−0.68	4.15	0	6	6	3.20	−10.28	−2.81	−10.00	1.05	4.43	3.73	1.96	0.19
**69**	**escitalopram**	1	−0.03	1	1	−2.37	0.56	3	0.42	1.00	0.52	0.95	0.45	3.24	0	3	3	2.57	−9.21	−8.44	−8.37	0.39	5.46	3.08	0.87	1.51
**70**	**estradiol benzoate**		−1.07	0	0		0.95	1	0.98	1.01	0.62	0.98		3.73	0	4	4	5.95	−10.11	−1.40	−9.97	2.51	4.77	3.58	4.20	0.84
**71**	**estrone**		−1.02	0	0	−3.05	0.95	1	0.99	1.00	0.77	0.97	1.04	4.02	1	6	7	5.76	−10.17	−2.21	−9.95	2.53	4.73	3.67	2.86	1.47
**72**	**ethambutol**	0	−0.49	1	1	−2.39	0.25	4	0.21	1.11	0.95	0.98	0.24	2.04	4	4	8	1.11	−9.11	23.39	−11.45	−2.21	4.95	2.18	0.29	
**73**	**ethanol**	1	0.22	1	1								−1.76	4.61	1	1	2					−0.19				
**74**	**famotidine**	1	−3.26	0	0	−0.73	0.175	4	0.71	1.01	0.99	1.01	0.00	3.36	8	9	17	1.01	−8.55	−5.85	−7.97	−1.02	4.85	2.69	−1.11	−0.58
**75**	**fexofenadine**	0	−0.75	1	0	−0.27	0.65	2	0.60	0.98	0.86	1.01	0.03	5.02	3	5	8	2.61	−8.98	−2.28	−8.75	2.3	6.27	4.87	3.81	0.03
**76**	**fluconazole**	1	−0.60	1	0	−3.81			0.87	1.00	0.89	0.99	−1.05	3.06	1	7	8	0.52	−10.28	−0.83	−10.19	0.5	3.44	2.49	0.84	−0.37
**77**	**fluoxetine**	1	0.21	1	1	−2.85	0.945	1	0.67	0.99	0.73	1.35	0.78	3.09	1	2	3	4.43	−9.41	−3.95	−9.02	1.56	4.99	2.71	1.93	1.82
**78**	**flupenthixol**	1	−0.29	1	1	−0.15	0.95	1	0.52	1.02	0.66	1.00	1.19	4.30	1	3	4	4.80	−7.61	−8.51	−6.76	4.42	5.85	3.65	5.33	1.71
**79**	**fluvoxamine**	1	−0.36	1	1	−2.19	0.79	2	0.32	0.94	0.62	1.00	−0.60	3.18	1	4	5	3.9	−9.69	−8.25	−8.87	1.15	5.89	2.79	2.69	
**80**	**furosemide**	0	−1.55	0	0	−4.16	0.95	1	0.92	1.00	0.96	1.00	1.07	3.11	4	7	11	3.27	−8.92	−10.23	−7.90	−0.12	4.19	2.42	−0.24	−0.56
**81**	**gabapentin**	1	−0.47	1	1	−0.29	0.03	4	0.91	1.00	0.66	1.00	−0.84	1.71	2	3	5	1.56	−9.35	8.36	−10.19	−1.31	2.38	1.7	0.96	
**82**	**gentamycin**	0	−2.65	0	0	−2.57	0.15	4	0.94	0.98	0.90	0.99	0.18	4.77	8	12	20	4.31	−9.34	18.62	−11.20	−7.81	4.71	4.25	−3.46	
**83**	**gliclazide**	1	−0.84	1	0	−1.13	0.94	1	0.95	1.00	0.80	0.96	0.99	3.23	2	6	8	5.31	−9.38	−10.80	−8.30	−0.28	4.91	2.86	1.90	−0.55
**84**	**haloperidol**	1	−0.10	1	1	−0.84	0.92	1	0.65	0.98	0.64	1.05	0.68	3.76	1	3	4	2.85	−9.22	−4.64	−8.75	2.11	5.29	3.37	1.31	1.38
**85**	**hydrochlorotiazide**	0	−1.35	0	0	−1.75	0.68	2	0.96	1.00	0.96	0.99	−0.22	2.97	3	5	8	7.27	−9.64	−11.83	−8.46	−0.09	3.85	2.01	−3.24	
**86**	**hydrocortisone acetate**		−0.42	1	1	−5.22	0.95	1	0.96	1.00	0.82	1.00	−0.18	3.73	0	4	4	4.23	−10.14	−0.86	−10.06	4.53	4.77	3.58	4.20	0.45
**87**	**hydroxyzine**	1	−0.03	1	1	−0.58	0.93	1	0.55	1.00	0.59	0.95	0.57	3.75	1	4	5	1.27	−8.81	−0.65	−8.75	2	5.48	3.54	1.23	1.14
**88**	**ibuprofen**	1	−0.05	1	1	−2.79	0.95	1	0.90	0.96	0.81	0.98	1.82	2.06	1	2	3	1.91	−9.39	1.92	−9.58	0.8	2.52	2.42	3.83	−0.07
**89**	**indomethacin**	1	−0.47	1	1	−3.24	0.97	1	0.90	1.02	0.87	0.99	1.81	3.58	1	5	6	2.98	−8.69	−5.75	−8.12	−0.16	4.94	3.05	−1.43	0.03
**90**	**ipratropium**	1	−0.20	1	1				0.43	1.00	0.54	1.00	−0.48	3.49	1	2	3	10.92	−12.36	−39.78	−8.39	−2.2	5.15	3.55	2.44	0.47
**91**	**isosorbide**	1	−0.95	0	0	−5.89			0.23	0.88	0.55	1.02	−0.74	1.91	1	6	7	2.81	−11.01	−1.93	−10.82	−0.51	2.88	1.43	−2.19	−0.68
**92**	**itraconazole**	0	−1.07	0	0	−0.73	1.00	1	0.97	1.01	0.84	0.98	0.98	7.06	0	9	9	6.11	−8.42	−7.7	−7.65	4.26	8.43	6.15	8.68	
**93**	**ketoprofen**	1	−0.32	1	1	−2.97	0.99	1	0.89	1.00	0.86	0.95	1.39	2.54	1	3	4	4.50	−9.96	−3.99	−9.56	−0.25	4.07	2.36	2.56	−0.71
**94**	**ketorolac**	1	−0.40	1	1	−2.91	0.99	1	0.91	0.99	0.96	1.00	0.98	2.59	1	3	4	2.14	−9.19	−3.63	−8.83	−0.95	3.87	2.39	1.79	

**95**	**ketotifen**	1	−0.23	1	1	−1.64	0.75	2	0.36	1.06	0.74	0.99	0.77	3.09	0	2	2	4.07	−9.08	−10.10	−8.07	3.28	3.89	2.87	0.26	1.34
**96**	**lamotrigine**	1	−0.90	1	0	−1.81	0.55	3	0.90	1.01	0.86	0.99	0.03	2.56	4	5	9	2.75	−8.22	−5.61	−7.66	−0.19	3.15	1.93	1.38	−0.15
**97**	**levetiracetam**	1	−0.47	1	1		0.10	4	0.92	1.00	0.92	1.00	−1.16	1.70	1	2	3	5.71	−9.8	6.49	−10.45	−0.67	3.09	1.62	−0.71	
**98**	**levocetrizine**	1	−0.30	1	1	−3.74	0.915	1	0.64	1.02	0.83	1.00	0.25	3.89	1	5	6	1.73	−9.00	−1.67	−8.83	−1.13	5.59	3.55	3.48	0.58
**99**	**levofloxacin**		−0.63	1	0	−2.01	0.31	3	0.17	0.97	0.30	1.11	0.60	3.61	1	7	8	7.21	−8.72	−8.37	−7.88	0.65	4.75	3.06	0.62	−0.04
**100**	**lincomycin**	0	−1.47	0	0	−1.58	0.70	2	0.91	1.00	0.94	1.00	−0.08	4.07	5	8	13	3.92	−8.5	0.04	−8.50	−0.48	6.35	3.89	0.48	
**101**	**loperamide**	0	−0.15	1	1	−0.56	0.97	1	0.67	0.99	0.77	1.03	0.65	4.77	1	4	5	4.65	−8.52	−0.23	−8.50	3.53	4.89	4.33	5.01	
**102**	**loratadine**	1	−0.13	1	1	−2.93	0.98	1	0.94	1.00	0.68	1.00	1.02	3.69	0	4	4	2.90	−9.02	−6.59	−8.36	5.94	4.93	3.31	0.30	1.07
**103**	**lorazepam**	1	−0.44	1	1	−3.6	0.87	2	0.94	1.00	0.83	1.00	0.34	3.21	2	4	6	6.40	−7.77	−1.17	−7.65	2.47	2.58	2.53	3.42	0.96
**104**	**medazepam**	1	0.30	1	1	−1.02	0.99	1	0.94	1.03	0.65	0.98	1.25	3.30	0	2	2	4.08	−8.34	−0.59	−8.28	4.42	2.80	2.49	4.29	1.54
**105**	**meloxicam**	0	−1.63	0	0	−2.7	0.99	1	0.98	0.99	0.95	1.00	1.80	3.51	2	7	9	5.35	−7.64	−12.59	−6.38	−0.09	4.03	2.71	−1.16	−0.31
**106**	**mesalazine**	0	−0.79	1	0	−5.30	0.43	3	0.93	0.99	0.97	0.98	−0.73	1.53	3	4	7	3.8	−8.18	−1.23	−8.06	−2.61	2.49	1.28	−1.76	
**107**	**metformin**	1	−0.88	1	0	−5.05			0.22	1.05	0.67	0.86	−0.17	129.60	4	5	9	1.58	−9.21	7.75	−9.99	−4.31	2.61	1.28	0.35	−0.47
**108**	**methyl paraben**		−0.20	1	1	−1.11			0.97	1.00	0.86	1.01	0.19	1.52	1	3	4	1.54	−9.53	−4.23	−9.11	1.81	3.08	1.35	−0.01	0.29
**109**	**methyldopa**	0	−1.11	0	0	−1.63	0.2	4	0.37	0.96	0.86	1.01	−0.44	2.11	5	5	10	1.56	−9.07	2.75	−9.34	−2.38	3.34	1.85	−1.46	−1.02
**110**	**metoclopramide**	1	−0.53	1	0	−1.88			0.29	1.00	0.64	0.98	0.39	3.00	3	5	8	3.80	−8.88	−2.89	−8.59	0.02	5.41	2.80	1.27	0.79
**111**	**metoprolol**	0	−0.26	1	1	−2.23			0.48	1.00	0.25	0.89	−0.06	3.09	2	4	6	1.53	−9.16	2.69	−9.43	0.034	7.00	3.22	1.57	0.64
**112**	**mianserin**	1	0.44	1	1	−1.06	0.9	2	0.55	1.01	0.55	1.00	0.81	2.64	0	2	2	0.56	−8.66	2.68	−8.93	2.76	3.65	2.64	0.94	1.34
**113**	**midazolam**	1	0.14	1	1	−1.17	0.97	1	0.83	1.01	0.66	1.00	0.51	3.26	0	3	3	3.03	−9.24	−1.28	−9.11	3.92	3.04	2.76	3.41	0.76
**114**	**minoxidil**	1	−0.95	0	0	−1.66			0.42	0.98	0.82	1.03	−0.40	2.08	2	5	7	4.2	−8.07	0.29	−8.10	0.65	3.16	1.96	0.97	
**115**	**mirtazapine**	1	0.24	1	1	−0.9	0.85	2	0.39	0.99	0.58	1.00	0.47	2.65	0	3	3	1.76	−8.79	0.19	−8.81	1.97	3.57	2.60	1.16	0.77
**116**	**montelukast**	1	−0.58	1	0	−2.44	0.99	1	0.95	1.00	0.69	0.99	−0.72	5.88	2	4	6	3.34	−7.46	−6.36	−6.83	5.19	7.27	5.47	4.13	2.00
**117**	**naproxen**	1	−0.20	1	1	−2.36	0.99	1	0.89	0.97	0.87	0.99	1.95	2.30	1	3	4	1.92	−9.10	−2.79	−8.82	0.47	3.89	2.15	2.99	−0.51
**118**	**nebivolol**	1	−0.59	1	0	−1.45	0.98	1	0.75	1.00	0.40	1.00	0.87	4.05	3	5	8	2.46	−9.05	−1.07	−8.94	2.4	5.05	3.53	−1.03	
**119**	**nitrendipine**	0	−1.22	0	0	−4.41	0.99	1	0.97	1.00	0.86	1.00	0.55	3.46	1	5	6	6.92	−9.09	−10.94	−8.00	3.50	5.3	2.98	−0.84	
**120**	**ofloxacin**	0	−0.63	1	0	−2.01	0.32	3	0.23	0.96	0.50	0.93	0.82	3.61	1	7	8	4.68	−8.72	−8.97	−7.82	−0.65	4.74	3.07	−2.61	
**121**	**olanzapine**		−0.80	1	0	−0.13	0.175	4	0.29	1.02	0.50	0.94	0.68	3.28	1	6	7	7.55	−9.20	−7.85	−8.41	−1.48	6.19	3.01	−1.56	1.31
**122**	**oxazepam**	1	−0.44	1	1	−3.74	0.85	2	0.94	1.01	0.84	1.01	0.22	2.87	2	4	6	3.38	−9.09	−1.06	−8.98	2.32	2.72	2.72	2.91	0.68
**123**	**oxcarbamazepine**	1	−0.47	1	1	−6.53	0.40	3	0.90	0.99	0.91	1.00	−0.40	2.52	1	2	3	3.46	−9.31	−4.73	−8.84	1.25	3.02	2.26	−0.41	
**124**	**oxybutinin**	1	−0.25	1	1	−1.04	0.92	1	0.66	0.97	0.24	0.83	0.60	3.57	1	3	4	1.02	−9.18	−0.66	−9.11	5.53	5.82	3.61	3.24	1.70
**125**	**PABA**		−0.47	1	1	−2.34			0.90	1.01	0.93	1.00	−0.82	1.37	3	3	6	4.29	−8.50	−2.10	−8.29	−1.61	2.54	1.22	−0.74	−1.88
**126**	**pantoprazole**	0	−0.83	1	0	−1.13	0.98	1	0.92	0.99	0.83	0.98	0.32	3.83	1	6	7	7.25	−9.09	−9.03	−8.19	1.5	5.08	3.01	−1.89	0.34
**127**	**paracetamol**	1	−0.24	1	1	−2.66	0.25	4	0.94	1.01	0.92	1.00	−0.55	1.51	2	3	5	3.28	−8.58	0.73	−8.65	0.34	3.03	1.39	−1.32	−0.49

**128**	**paroxetine**	1	−0.09	1	1	−2.48	0.94	1	0.56	1.02	0.67	1.12	0.78	3.31	1	4	5	2.12	−9.00	0.34	−9.04	1.19	4.12	2.99	−0.49	2.06
**129**	**pefloxacin**	0	−0.48	1	1	−7.04	0.25	4	0.29	0.97	0.70	0.92	1.09	3.33	1	6	7	9.41	−9.01	−10.45	−7.97	0.52	4.66	2.91	−1.54	
**130**	**pergolide**	1	−0.16	1	1	−0.78	0.9	2	0.47	0.98	0.53	0.96	−1.15	3.14	1	2	3	1.93	−8.35	1.51	−8.50	2.29	4.50	3.09	2.29	
**131**	**perindopril**	0	−0.99	0	0	−4.05	0.15	4	0.46	0.97	0.58	1.02	−0.31	3.68	2	7	9	6.26	−9.78	2.88	−10.07	−0.01	5.43	3.52	2.06	−0.76
**132**	**phenytoin**	1	−0.38	1	1	−0.88	0.9	2	0.97	1.00	0.86	0.99	0.21	2.52	2	4	6	2.98	−9.84	−2.94	−9.55	2.48	3.32	2.26	0.73	0.25
**133**	**pindolol**	1	−0.37	1	1	−2.34	0.4	3	0.52	0.98	0.78	1.03	0.22	2.48	3	3	6	3.19	−8.47	0.63	−8.53	0.19	4.60	2.44	−1.23	0.78
**134**	**piroxicam**	0	−1.18	0	0	−2.7	0.99	1	0.98	1.00	0.60	1.03	1.52	3.33	2	7	9	11.24	−8.63	2.05	−8.84	−2.14	2.66	2.50	1.07	−0.79
**135**	**prednizolone**	1	−0.97	0	0	−5.26	0.9	2	0.94	0.99	0.88	1.01	−0.28	3.46	3	5	8	3.68	−10.01	−4.70	−9.54	1.49	3.96	3.14	2.01	0.45
**136**	**pregabalin**	1	−0.47	1	1	−0.57			0.76	1.00	0.41	1.00	−0.87	1.59	2	3	5	1.67	−9.53	9.29	−10.46	−1.38	3.54	1.65	0.89	
**137**	**primidone**	1	−0.38	1	1	−5.06	0.70	2	0.93	1.03	0.97	1.04	−0.66	2.18	2	2	4	2.84	−9.78	−0.15	−9.77	0.40	3.19	2.02	0.72	
**138**	**progesterone**		−0.20	1	1		0.975	1	0.96	1.00	0.68	0.96	0.76	3.77	1	3	4	5.88	−9.29	−2.60	−9.03	6.24	4.54	3.60	3.12	1.54
**139**	**promazine**	1	0.04	1	1	−2.23	0.94	1	0.37	0.95	0.51	1.00	0.95	2.84	0	2	2	2.86	−7.54	−3.93	−7.15	2.67	2.93	2.53	3.25	−1.02
**140**	**promethazine**	1	0.04	1	1	−1.78	0.93	1	0.42	0.98	0.47	0.96	0.73	2.84	0	2	2	3.40	−7.83	−0.65	−7.76	3.2	4.36	2.76	−0.20	1.17
**141**	**propafenone**	0	−0.39	1	1	−2.11	0.97	1	0.59	1.04	0.61	1.09	0.51	3.29	2	4	6	2.97	−8.77	−1.75	−8.59	2.06	5.36	3.28	1.86	1.82
**142**	**propranolol**	1	−0.12	1	1	−2.3	0.9	2	0.50	0.93	0.63	0.98	0.54	2.59	2	3	5	1.53	−8.36	−5.71	−7.79	1.27	3.09	2.56	2.80	1.45
**143**	**propylthiouracil**	1	−0.11	1	1	−0.43	0.82	2	0.94	1.00	0.93	1.00	−0.48	1.70	2	1	3	5.57	−9.2	−12.71	−7.93	1.18	3.32	1.51	0.71	
**144**	**pseudoephedrine**	1	0.03	1	1	−2.18			0.89	1.00	0.56	1.04	−0.06	1.65	2	2	4	2.62	−9.25	5.04	−9.75	−0.9	3.30	1.71	0.85	0.34
**145**	**quetiapine**	1	−0.63	1	0	−0.46	0.83	2	0.68	1.00	0.69	0.95	0.39	3.84	1	5	6	1.57	−8.68	−7.24	−7.95	1.55	5.42	3.55	2.84	1.23
**146**	**quinapril**	0	−0.99	0	0	−3.81	0.97	1	0.44	1.00	0.56	0.98	0.49	4.39	2	5	7	5.13	−9.50	0.91	−9.59	0.85	6.10	4.10	1.69	0.09
**147**	**quinine**	1	−0.18	1	1	−2.08	0.70	2	0.29	0.97	0.64	0.97	0.41	3.14	1	4	5	1.73	−9.55	−0.14	−9.54	1.58	4.22	3.06	1.2	
**148**	**ranitidine**	0	−1.24	0	0	−1.15			0.24	1.00	0.80	0.99	0.07	3.14	2	7	9	8.02	−9.24	−7.19	−8.52	0.19	6.30	2.89	−1.18	−0.17
**149**	**rifampicin**	0	−2.98	0	0	−2.24	0.89	2	0.77	1.05	0.94	0.99	−1.53	8.23	6	14	20					−0.46				
**150**	**rimantadine**	1	0.13	1	1	−3.56	0.40	3	0.92	1.00	0.95	1.00	−0.80	1.79	1	1	2	1.22	−9.33	29.54	−12.28	0.08	2.43	1.91	2.19	
**151**	**risperidone**	1	−0.44	1	1	−0.87	0.88	2	0.29	0.98	0.58	0.94	0.32	4.10	0	6	6	3.42	−8.93	−8.76	−8.06	2.27	4.91	3.73	0.63	0.68
**152**	**rizatriptan**	1	−0.40	1	1	−2.29	0.14	4	0.20	0.98	0.77	1.07	0.31	3.12	1	4	5	1.76	−8.14	−6.90	−7.45	2.68	4.26	2.90	−0.27	0.40
**153**	**rosuvastatin**	0	−1.71	0	0	−2.95	0.88	2	0.89	0.99	0.88	0.96	0.29	4.82	3	8	11	5.66	−9.19	−10.63	−8.13	−2.63	6.89	4.12	1.18	−0.02
**154**	**roxitromicin**	0	−2.92	0	0	−0.96	0.96	1	0.92	1.01	0.79	0.99	0.01		5	16	21				0.00	2.9			2.90	
**155**	**rupatadine**		0.08	1	1	−0.25	0.985	1	0.43	1.02	0.52	0.95	1.09	4.15	0	3	3	1.61	−8.99	−6.43	−8.35	5.97	4.97	3.94	1.63	0.97
**156**	**sertraline**	1	0.35	1	1	−2.27	0.98	1	0.53	1.00	0.48	0.98	1.12	3.06	1	1	2	2.87	−9.19	−0.94	−9.09	2.77	4.21	2.72	1.68	1.63
**157**	**sildenafil**	1	−1.20	0	0	−1.17	0.96	1	0.67	0.97	0.76	0.99	0.21	4.75	1	8	9	5.84	−8.91	9.47	−9.86	2.27	6.47	4.20	−1.47	1.15
**158**	**simvastatin**	1	−0.62	1	0	−6.29	0.95	1	0.17	0.89	0.65	0.98	0.94	4.19	1	5	6	5.78	−9.10	2.25	−9.33	4.41	5.71	4.10	4.43	1.12
**159**	**sotalol**	1	−0.84	1	0	−2.11			0.46	0.95	0.85	1.00	−0.01	2.72	3	5	8	5.15	−9.09	−5.13	−8.58	−1.45	5.19	2.48	−0.45	0.13
**160**	**spironolactone**	1	−0.82	1	0		0.9	2	0.96	0.97	0.77	0.99	−0.09	4.17	0	4	4	1.67	−9.76	−7.98	−8.96	3.12	4.94	3.82	3.33	1.13

**161**	**sulpiride**	1	−1.21	0	0	−1.77	0.4	3	0.26	0.96	0.86	1.01	0.16	3.41	3	7	10	3.65	−7.89	−16.74	−6.22	−1.12	5.08	3.02	0.48	0.22
**162**	**telmisartan**	1	−0.46	1	1	−3.34	0.995	1	0.90	0.99	0.85	0.98	1.35	5.01	1	6	7	0.71	−8.56	−5.48	−8.02	4.76	6.39	4.69	7.39	0.67
**163**	**temazepam**	1	−0.30	1	1	−4.46	0.96	1	0.94	0.99	0.79	1.00	0.10	3.01	1	4	5	2.48	−9.17	−0.83	−9.09	2.15	2.84	2.53	3.15	0.83
**164**	**theophylline**	1	−0.56	1	0	−1.4	0.4	3	0.87	1.01	0.93	0.99	−0.66	1.80	1	6	7	3.47	−9.10	−5.77	−8.53	−0.2	3.02	1.50	−1.31	−0.94
**165**	**thioridazine**	1	−0.37	1	0	−2.64	0.95	1	0.43	0.91	0.40	1.00	1.27	3.71	0	2	2	0.98	−7.92	−2.68	−7.65	3.94	4.89	3.50	4.18	1.68
**166**	**timolol**	0	−0.73	1	0	−2.15	0.10	4	0.32	0.94	0.97	1.00	−0.88	3.16	2	7	9	3.06	−9.17	−11.24	−8.05	−1.39	4.95	2.88	1.21	
**167**	**tinidazole**	1	−1.02	0	0	−4.90	0.12	4	0.88	0.99	0.93	0.99	−0.92	2.47	0	5	5	2.34	−10.52	−12.82	−9.24	−0.27	4.41	2	0.03	
**168**	**tolterodine**	1	0.17	1	1	−3.48	0.963	1	0.08	0.80	0.43	0.96	0.46	3.25	1	2	3	2.18	−8.95	2.20	−9.17	2.94	5.73	3.44	3.24	−0.68
**169**	**tramadol**	1	0.02	1	1	−2.41	0.20	3	0.31	1.00	0.64	1.02	0.03	2.63	1	3	4				0.00	0.36				
**170**	**trazodone**	1	−0.13	1	1	−0.32	0.92	1	0.72	1.01	0.74	0.97	0.35	3.72	0	6	6	3.41	−8.50	−6.21	−7.88	1.58	5.01	3.36	2.80	0.97
**171**	**trimethoprim**	1	−1.14	0	0	−0.30	0.44	3	0.59	1.04	0.83	0.99	−0.07	2.90	2	7	9	2.09	−8.8	−1.22	−8.68	0.58	4.14	2.63	−2.47	
**172**	**tropicamid**	1	−0.31	1	1	−1.88	0.45	3	0.86	1.01	0.83	1.00	−0.82	2.84	1	3	4	4.06	−9.68	−2.81	−9.40	1.15	4.15	2.76	−0.01	0.03
**173**	**valproic acid**	1	−0.05	1	1	−2.38	0.85	2	0.67	0.97	0.04	1.00	0.65	1.44	1	2	3	1.93	−11.19	10.01	−12.19	0.16	3.75	5.54	2.61	−1.09
**174**	**valsartan**	0	−1.25	0	0	−3.64	0.955	1	0.91	1.02	0.96	0.99	1.69	4.22	2	8	10	2.92	−9.69	−6.39	−9.05	0.05	5.46	3.92	3.88	−1.10
**175**	**venlafaxine**	1	0.02	1	1	−2.07	0.27	4	0.37	1.03	0.65	1.07	0.02	2.77	1	3	4	3.25	−9.02	2.73	−9.29	1.07	4.44	2.85	1.44	0.92
**176**	**verapamil**	1	−0.48	1	1	−1.77	0.9	2	0.53	0.97	0.86	1.00	0.35	4.55	0	6	6	5.44	−8.76	0.90	−8.85	2.33	7.51	4.53	0.57	1.08
**177**	**warfarin**	1	−0.47	1	1	−2.70	0.99	1	0.99	1.00	0.83	0.95	1.14	3.08	1	3	4	4.12	−7.38	−13.35	−6.05	−0.90	4.01	2.76	0.46	
**178**	**zolmitriptane**	1	−0.37	1	1	−2.32	0.25	4	0.29	0.94	0.86	1.00	0.23	2.89	2	5	7	4.09	−8.73	−1.57	−8.57	−0.44	4.68	2.74	−1.01	0.52
**179**	**zolpidem**	1	−0.05	1	1	−0.43	0.92	1	0.88	1.02	0.77	1.01	0.25	3.07	0	4	4	6.69	−8.37	−0.45	−8.33	3.02	3.33	2.94	0.86	0.42
**180**	**zopiclone**	0	−0.92	0	0	−0.5	0.45	3	0.96	1.00	0.83	0.99	−0.12	3.89	0	9	9	5.47	−9.13	−1.78	−8.95	−42	3.36	3.86	2.31	−0.04
**181**	**zuclopenthixol**	1	−0.29	1	1	−0.17	0.985	1	0.47	1.00	0.60	0.97	1.21	3.97	1	3	4	2.45	−7.57	−4.53	−7.12	5.06	5.58	3.55	4.97	1.69

** – multiplied by 10

* – divided by 100

Table S7. The structures of 181 APIs

1. acebutolol

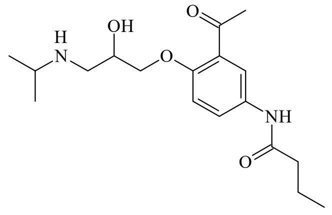

2. aceclofenac

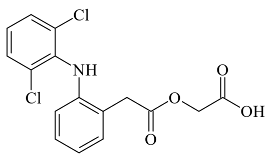

3. acenocumarol

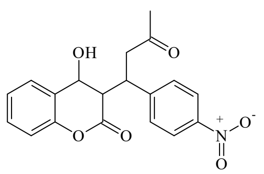

4. acetazolamid

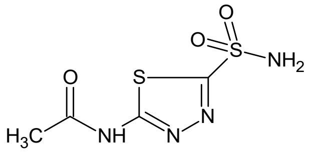

5. acetylsalicylic acid

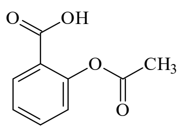

6. acyclovir

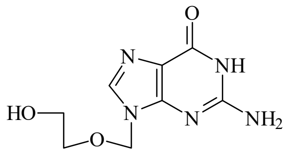

7. allopurinol

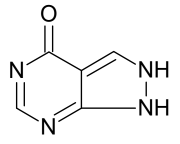

8. alprazolam

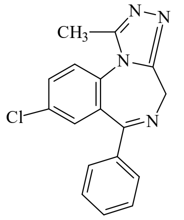

9. amiodarone

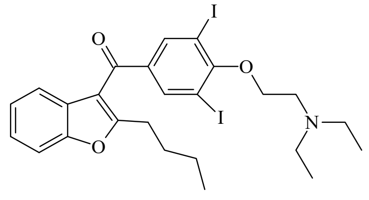

10. amitriptyline

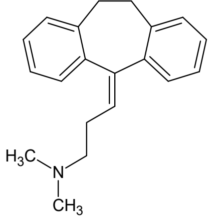

11. amlodipine

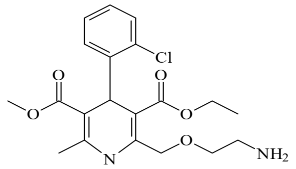

12. amoxilcillin

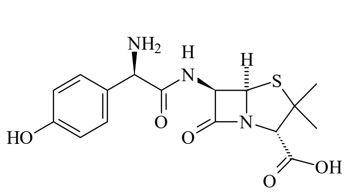

13. astemizol

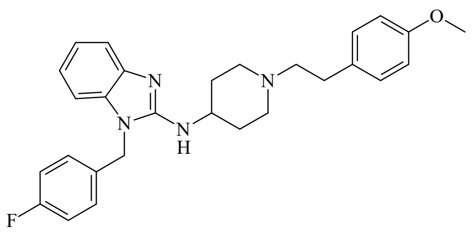

14. atenolol

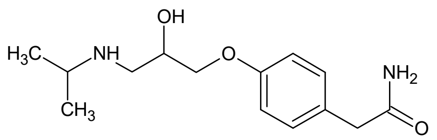

15. atorvastatin

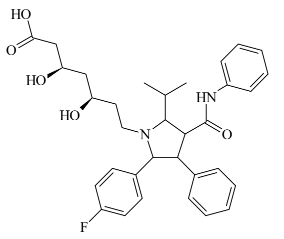

16. atropine

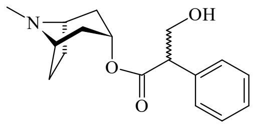

17. azithromycin

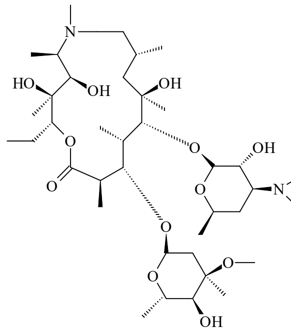

18. betahistine

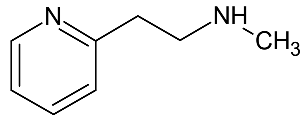

19. betaxolol

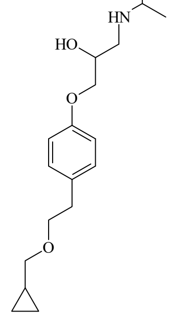

20. bilastine

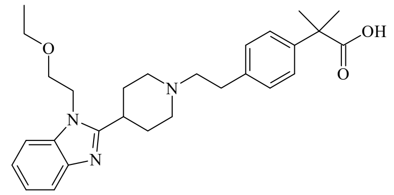

21. biperiden

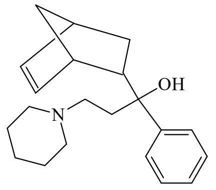

22. bisoprolol

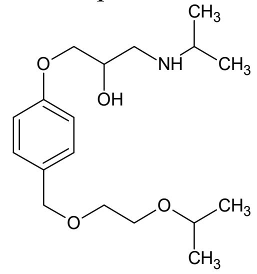

23. bromazepam

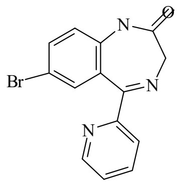

24. bromocriptine

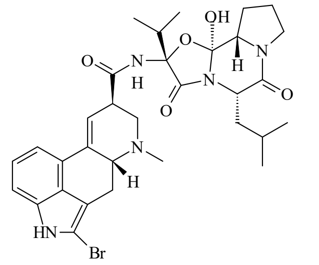

25. bupivacaine

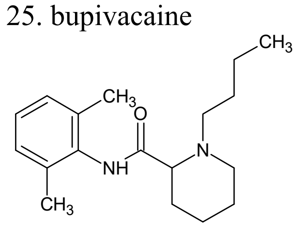

26. buspirone

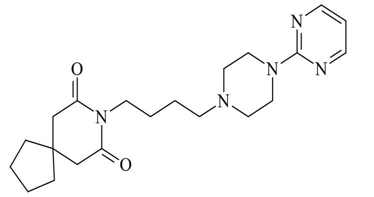

27. caffeine

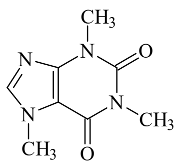

28. capecitabine

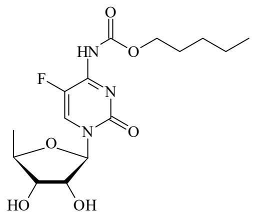

29. captopril

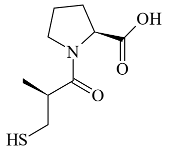

30. carbamazepine

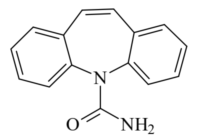

31. carbegoline

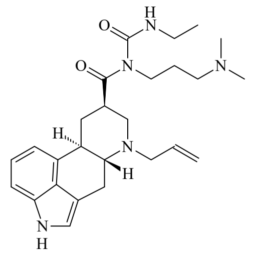

32. carvedilol

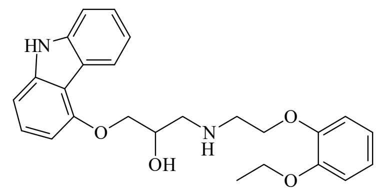

33. cefuroxime

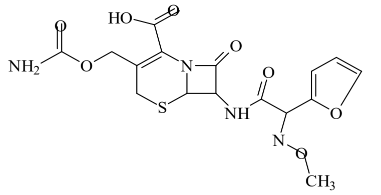

34. celecoxib

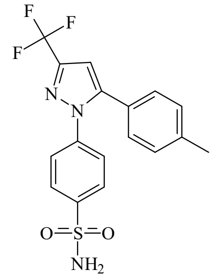

35. celiprolol

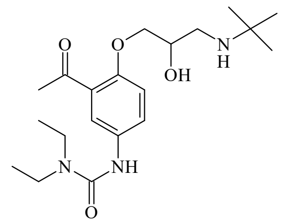

36. cephalexin

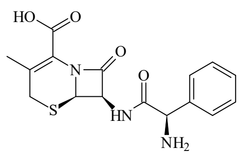

37. cetrizine

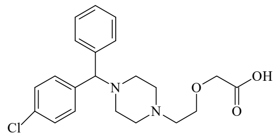

38. chloramphenicol

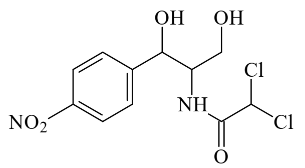

39. chloroquine

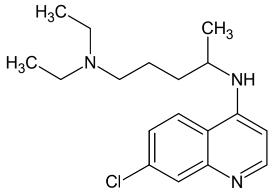

40. chlorpromazine

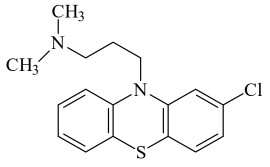

41. chlortalidone

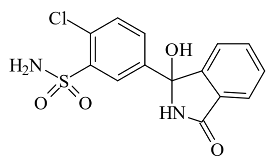

42. cimetidine

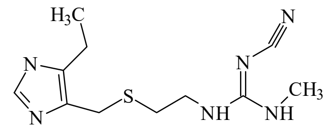

43. ciprofloxacin

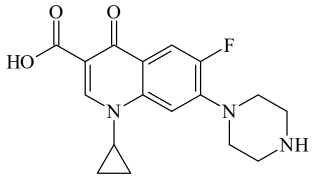

44. cisapride

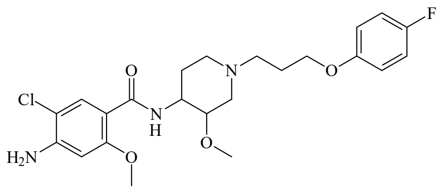

45. citalopram

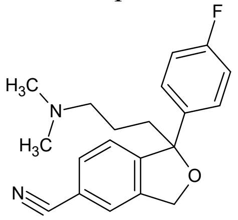

46. clarithromycin

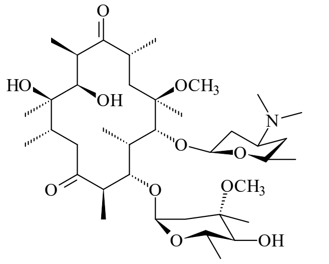

47. clindamycin

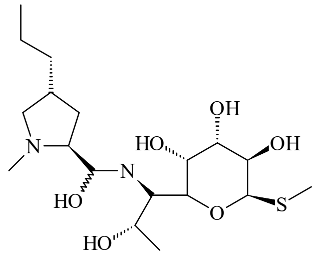

48. clobazam

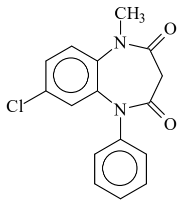

49. clomipramine

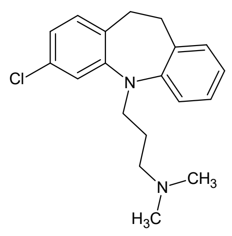

50. clonidine

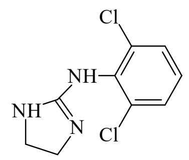

51. clorazepate

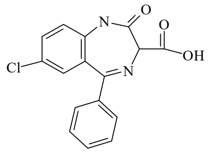

52. clozapine

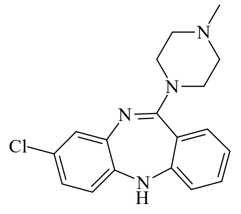

53. colchicine

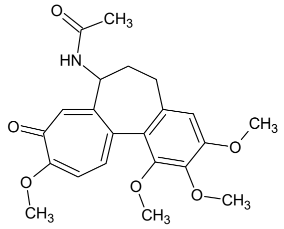

54. cyproheptadine

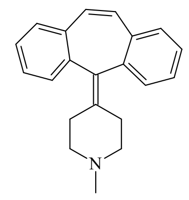

55. desloratidine

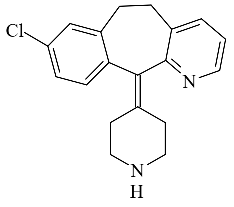

56. diazepam

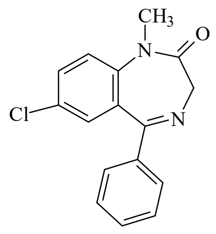

57. digoxin

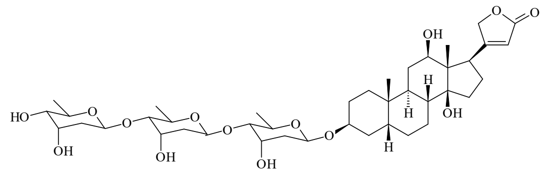

58. dihydroergotamine

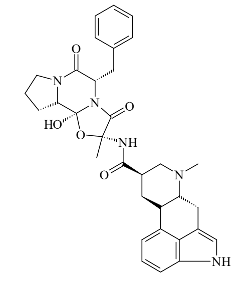

59. diltiazem

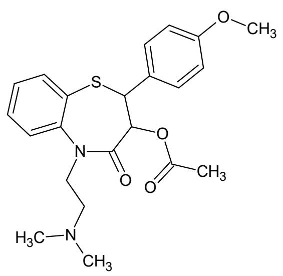

60. diphenhydramin

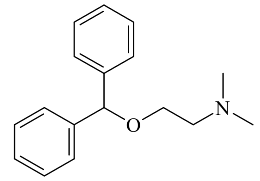

61. doxazosin

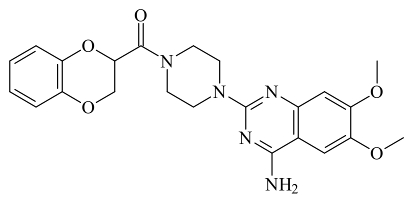

62. doxepin

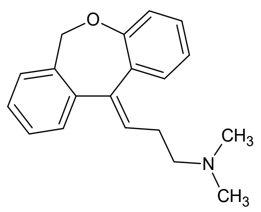

63. doxycycline

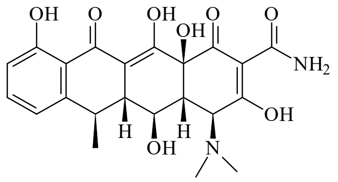

64. drotaverine

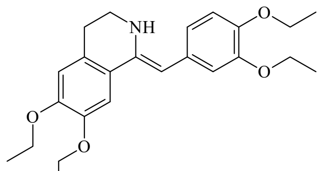

65. duloxetine

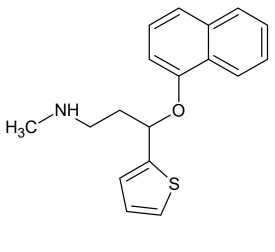

66. eletriptan

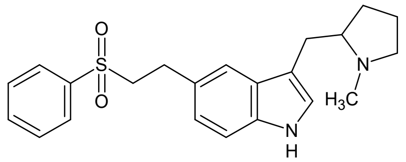

67.enalapril

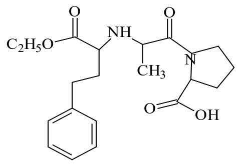

68. eplerenone

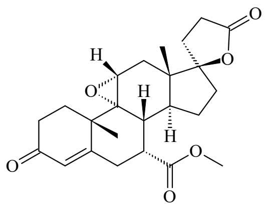

69. escitalopram

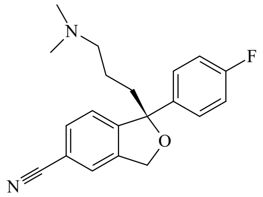

70. estradiol benzoate

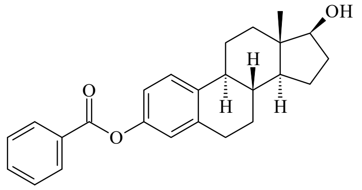

71. estrone

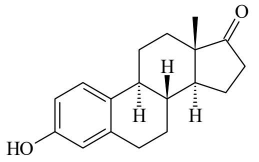

72. ethambutol

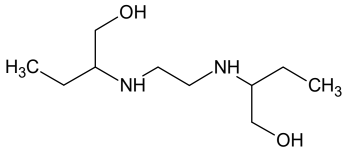

73. ethanol

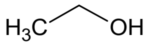

74. famotidine

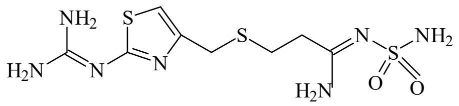

75. fexofenadine

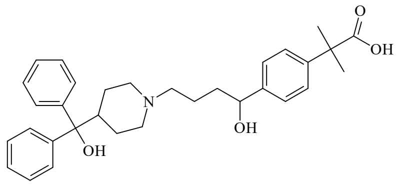

76. fluconazole

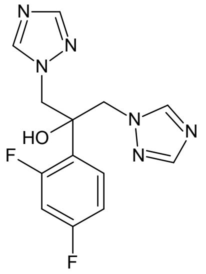

77. fluoxetine

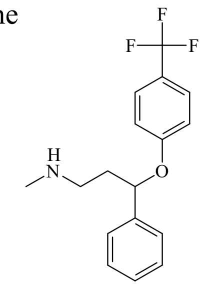

78. flupenthixol

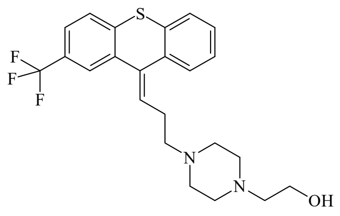

79. fluvoxamine

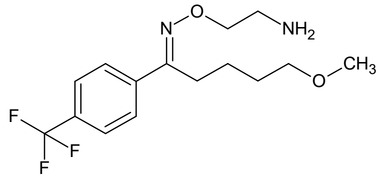

80. furosemide

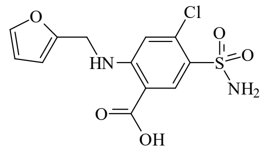

81. gabapentin

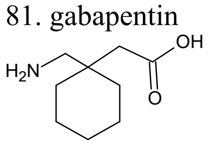

82. gentamycin

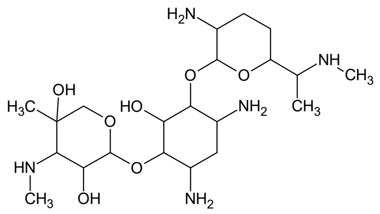

83. gliclazide

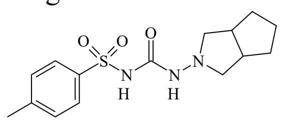

84. haloperidol

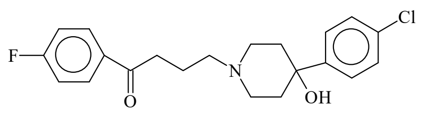

85. hydrochlorotiazide

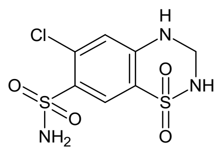

86. hydrocortisone acetate

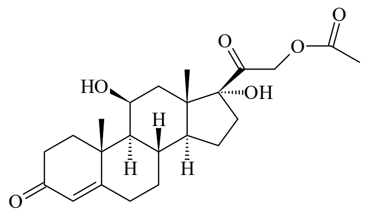

87. hydroxyzine

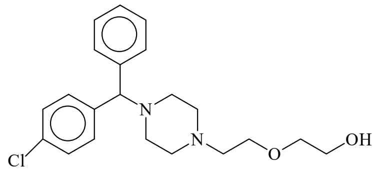

88. ibuprofen

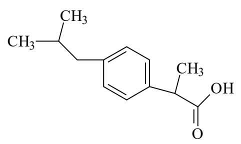

89. indomethacin

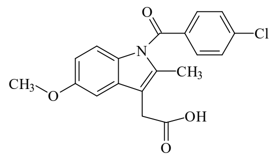

90. ipratropium

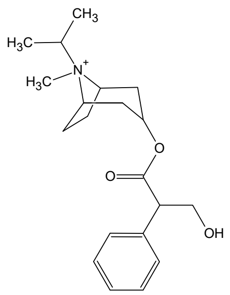

91. isosorbide

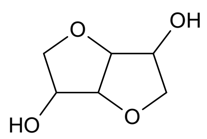

92. itraconazole

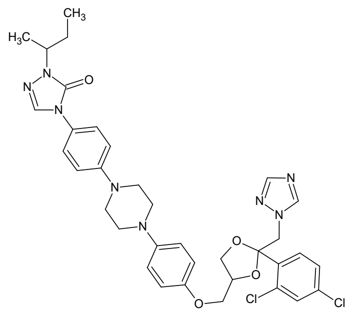

93. ketoprofen

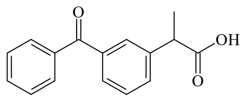

94. ketorolac

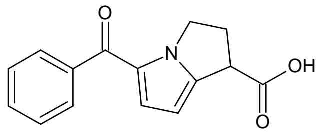

95. ketotifen

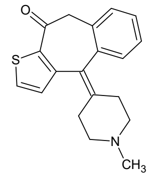

96. lamotrigine

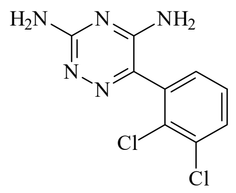

97. levetiracetam

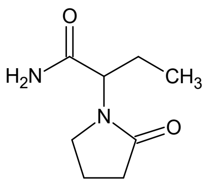

98. levocetrizine

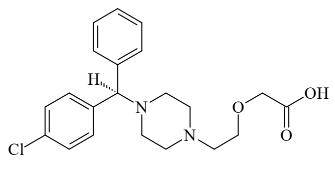

99. levofloxacin

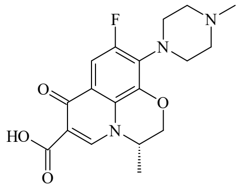

100. lincomycin

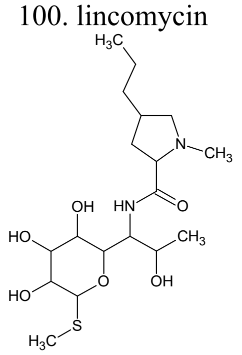

101. loperamide

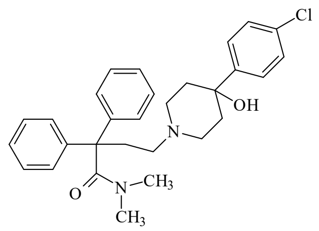

102. loratadine

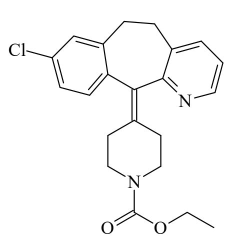

103. lorazepam

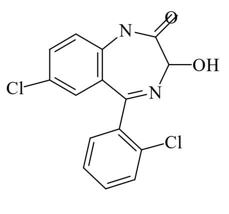

104. medazepam

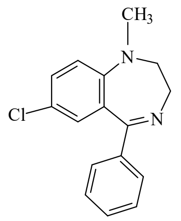

105. meloxicam

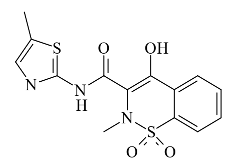

106. mesalazine

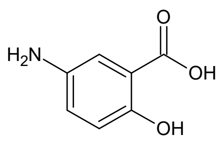

107. metformin

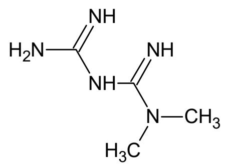

108. methyl paraben

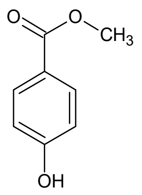

109. methyldopa

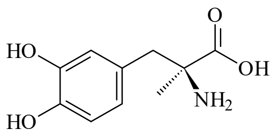

110. metoclopramide

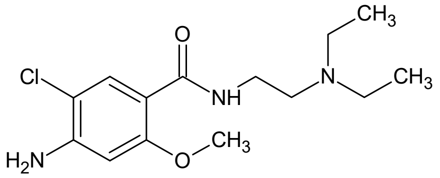

111. metoprolol

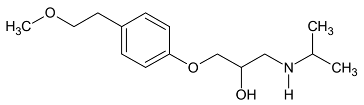

112. mianserin

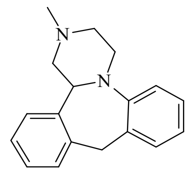

113. midazolam

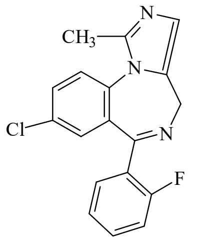

114. minoxidil

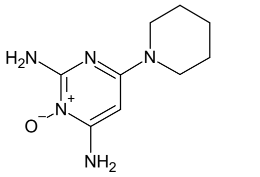

115. mirtazapine

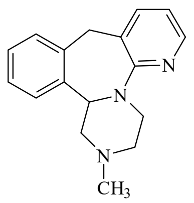

116. montelukast

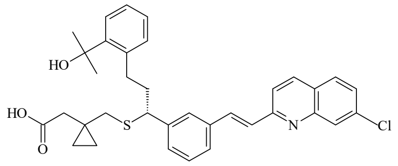

117. naproxen

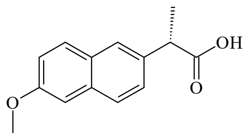

118. nebivolol

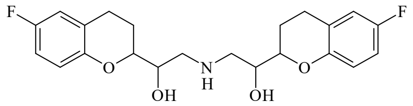

119. nitrendipine

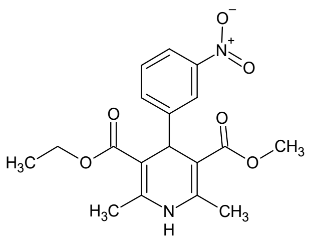

120. ofloxacin

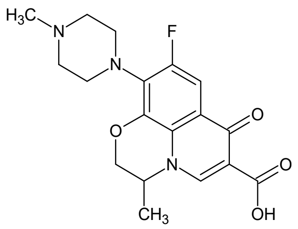

121. olanzapine

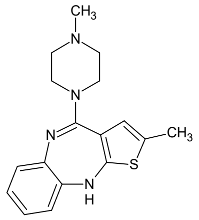

122. oxazepam

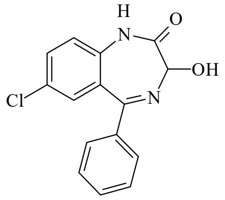

123. oxcarbamazepine

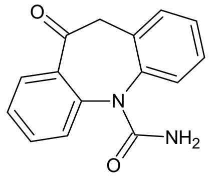

124. oxybutynin

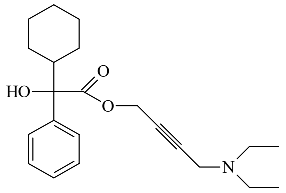

125. PABA

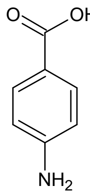

126. pantoprazole

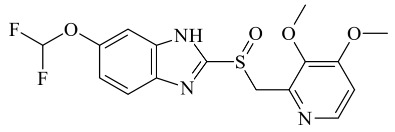

127. paracetamol

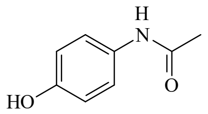

128. paroxetine

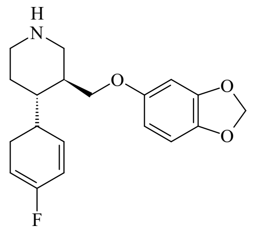

129. pefloxacin

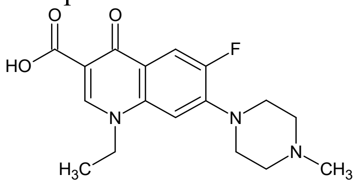

130. pergolide

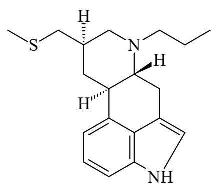

131. perindopril

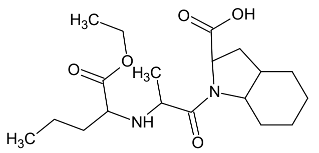

132. phenytoin

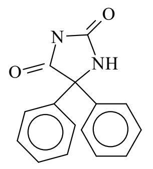

133. pindolol

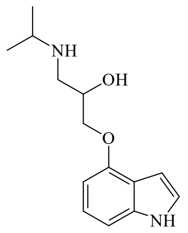

134. piroxicam

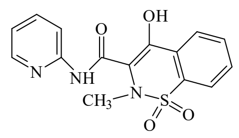

135. prednizolone

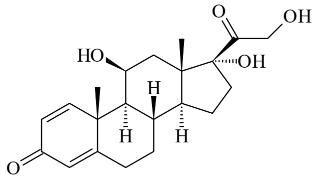

136. pregabalin

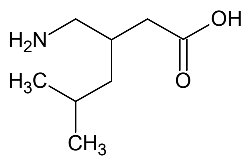

137. primidone

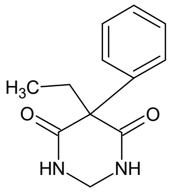

138. progesterone

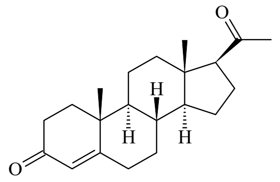

139. promazine

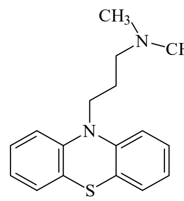

140. promethazine

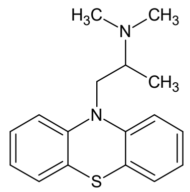

141. propafenone

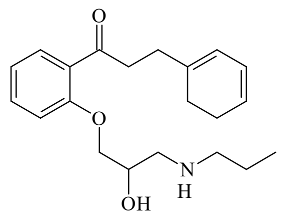

142. propranolol

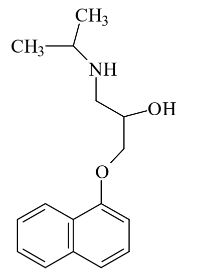

143. propylthiouracil

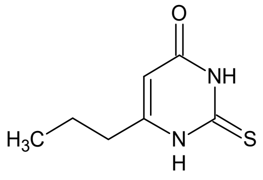

144. pseudoephedrine

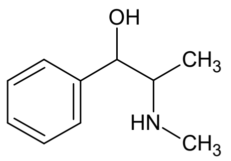

145. quetiapine

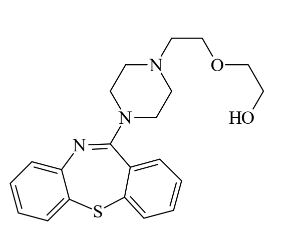

146. quinapril

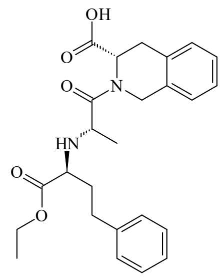

147. quinine

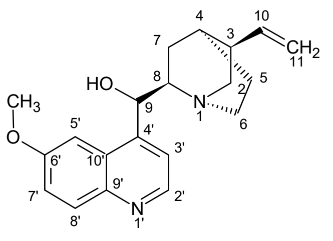

148. ranitidine

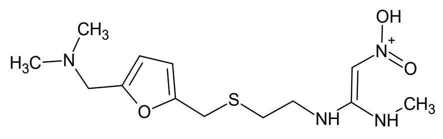

149. rifampicin

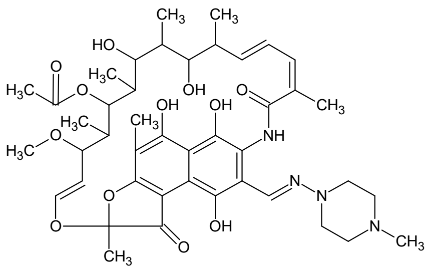

150. rimantadine

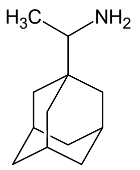

151. risperidone

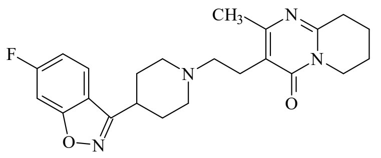

152. rizatriptan

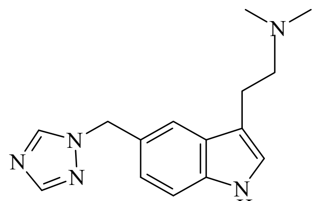

153. rosuvastatin

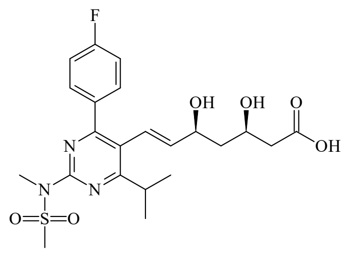

154. roxitromicin

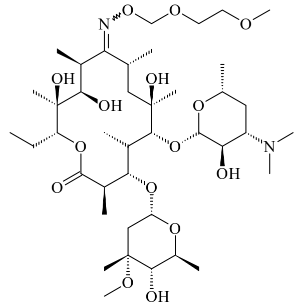

155. rupatadine

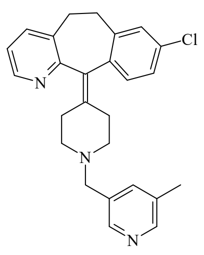

156. sertraline

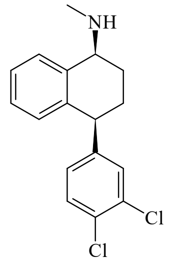

157. sildenafil

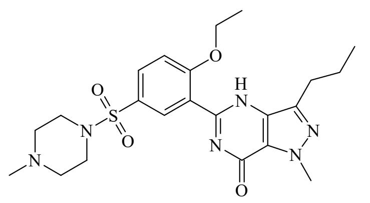

158. simvastatin

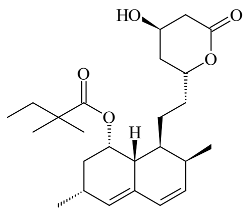

159. sotalol

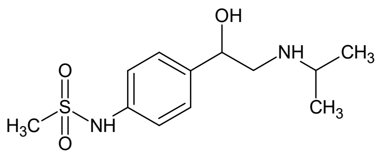

160. spironolactone

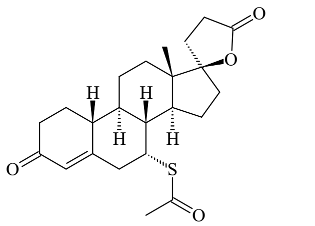

161. sulpiride

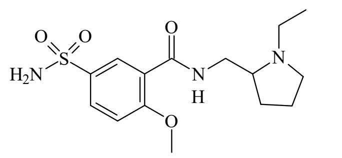

162. telmisartan

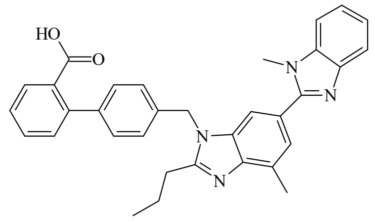

163. temazepam

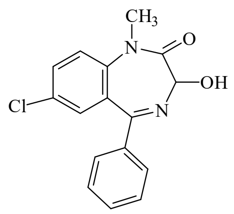

164. theophylline

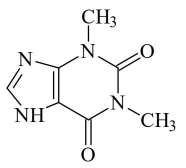

165. thioridazine

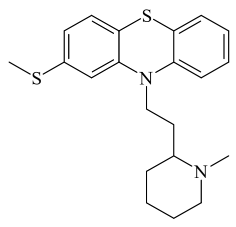

166. timolol

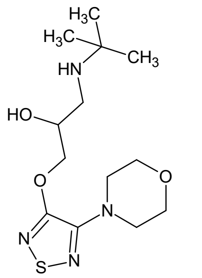

167. tinidazole

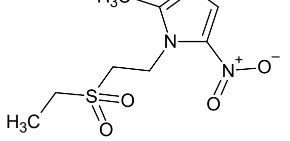

168. tolterodine

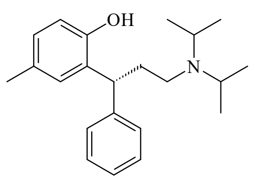

169.tramadol

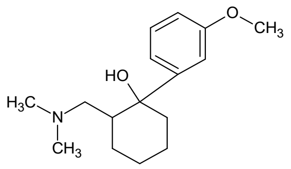

170. trazodone

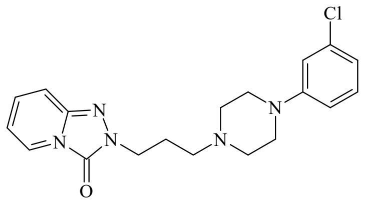

171. trimethoprim

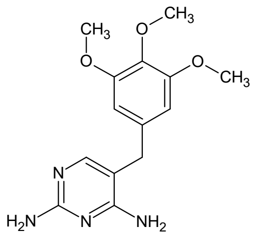

172. tropicamid

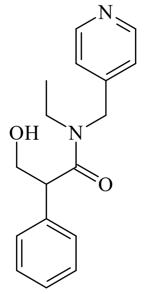

173. valproic acid

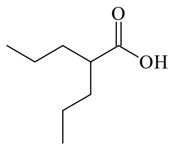

174. valsartan

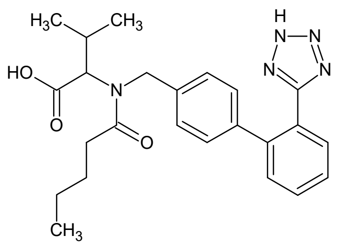

175. venlafaxine

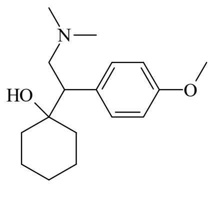

176. verapamil

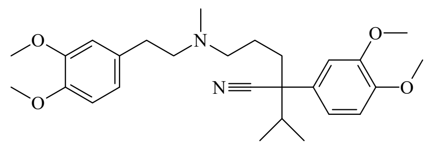

177. warfarin

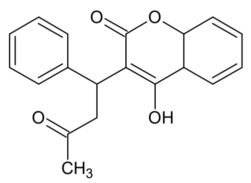

178. zolmitriptane

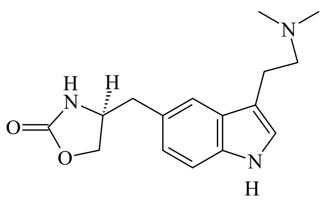

179. zolpidem

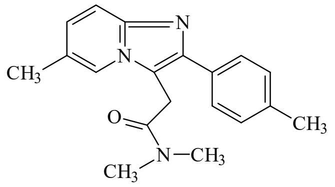

180. zopiclone

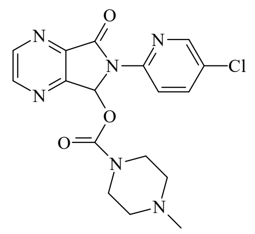

181. zuclopenthixol

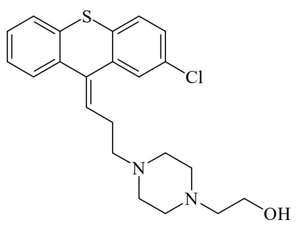

Supplement: Supplementary file 1 [file pharmaceutics-16-01534-s001.zip › pharmaceutics-3317296-supplementary.pdf]
